# Synthesis and Photocatalytic sp^3^ C-H Bond Functionalization of Salen-Ligand-Supported Uranyl(VI) Complexes

**DOI:** 10.3390/molecules29174077

**Published:** 2024-08-28

**Authors:** Jialu He, Xingxing Gong, Yafei Li, Qianyi Zhao, Congqing Zhu

**Affiliations:** 1 State Key Laboratory of Coordination Chemistry, Jiangsu Key Laboratory of Advanced Organic Materials, School of Chemistry and Chemical Engineering, Nanjing University, Nanjing 210023, China; jialuhe1228@163.com (J.H.); dg21240032@smail.nju.edu.cn (X.G.); 17865187280@163.com (Y.L.); 2 School of Chemistry and Chemical Engineering, Henan Normal University, Xinxiang 453007, China; qyzhao@htu.edu.cn

**Keywords:** photocatalysis, uranyl(VI), Salen ligand, uranium, sp^3^ C-H bond, C-H bond activation, uranyl-Salen complexes

## Abstract

Recent years have seen increasing interest in uranyl(VI) photocatalysis. In this study, uranyl complexes were successfully synthesized from ligands **L1**–**L6** and UO_2_(NO_3_)_2_·6H_2_O under reflux conditions, yielding products **1**–**6** with yields ranging from 30% to 50%. The complexes were thoroughly characterized using NMR spectroscopy, single-crystal X-ray diffraction, and elemental analysis. The results indicate that complexes **1**–**5** possess a pentagonal bipyramidal geometry, whereas complex **6** exhibits an octahedral structure. The photocatalytic properties of these novel complexes for sp^3^ C-H bond functionalization were explored. The results demonstrate that complex **4** functions as an efficient photocatalyst for converting C-H bonds to C-C bonds via hydrogen atom transfer under blue light irradiation.

## 1. Introduction

In recent years, photocatalysis under visible light has been recognized as an invaluable approach for environmentally sustainable organic synthesis [1,2,3,4,5,6,7,8,9,10,11,12,13,14,15,16,17,18,19,20,21,22,23]. These methodologies leverage light—an accessible, eco-friendly, and inexhaustible energy source—to drive reactions at room temperature and under mild conditions. Organic compounds typically contain C-H bonds, classified as sp, sp^2^, or sp^3^, depending on the p orbital’s involvement in their hybridization. Following the groundbreaking efforts by Heck and co-workers in the late 1960s on palladium-catalyzed coupling reactions [24], remarkable advancements have been achieved in the functionalization of the less reactive sp^2^ C-H bonds. However, the more abundant sp^3^ C-H bonds remain underutilized due to their low reactivity, strong bond dissociation energies, and significant thermodynamic stability [25,26,27,28,29,30]. Additionally, forming C-C bonds is a fundamental aspect of synthetic chemistry, often requiring the activation of a C-H bond, a process complicated in saturated hydrocarbons like cycloalkanes and ethers (e.g., THF and dioxopentane) by the lack of reactive functional groups.

The uranyl(VI) ion UO_2_ [31,32,33,34,35,36,37,38,39,40,41] is distinguished by its exceptional chemical stability. Its coordination chemistry is primarily constrained within the equatorial plane, attributed to the robust covalent nature of the axial U=O bonds. This configuration allows for four to six atoms to be symmetrically positioned on the equatorial plane, almost perpendicular to the O=U=O unit [42]. The photoexcitation of the uranyl ion leads to a highly oxidized state, typically characterized by a potential of around +2.6 V, and a notable lifetime of several microseconds. This state can be achieved through visible and ultraviolet light irradiation, within the 300–400 nm range. The subsequent ligand-to-metal charge transfer (LMCT) process is believed to generate highly active excited states of uranyl ions, which are prone to quenching upon interaction with organic hydrocarbons [43,44]. Quenching mechanisms include hydrogen-stripping reactions between uranyl ions and organic radicals from aliphatic groups and interception of these radicals by oxygen molecules, resulting in electron transfer to unsaturated or aromatic groups [45,46,47,48]. Despite the significant photochemical reactivity of uranyl ions, their exploration has predominantly focused on photophysical and redox properties, with their photocatalytic applications receiving less attention.

**Figure 1:**
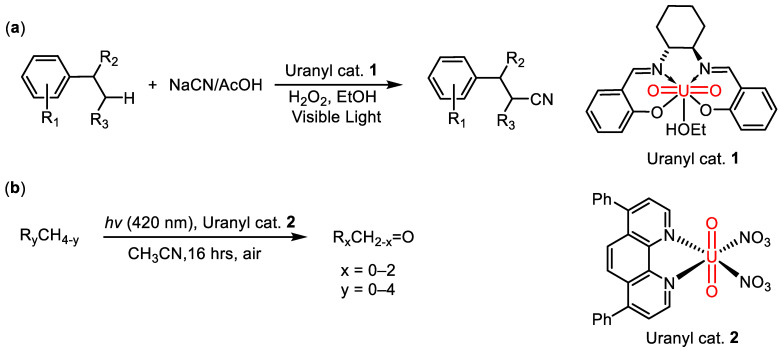
(**a**) Photocatalytic cyanation of sp^3^ C-H bonds utilizing a uranyl cat. **1**. (**b**) Photocatalytic oxidation of hydrocarbons by uranyl cat. **2**.

Recent explorations of uranyl complexes have broadened into catalytic utilities, including fluorination of C-H bonds, addition reactions, alkynylation, alkenylation, and oxidation processes [49,50,51,52,53,54,55,56,57,58,59,60,61,62,63]. Azam and colleagues [64] illustrated the use of a chiral Salen ligand to coordinate the uranyl’s equatorial plane, creating a photoactive complex that acts as an α-cyanation catalyst for aniline—a reactivity not observed with commercially available uranyl acetate (Figure 1a). Furthermore, the Arnold group [65] highlighted the activation of C-H bonds by a uranyl complex [U^VI^O_2_(NO_3_)_2_(phen)] (phen = phenanthrene) under light excitation. The novel uranyl photocatalyst U^Ph2phen^ showed superior conversion rates in oxidizing selected substrates, an improvement attributed to ligand-induced electronic effects (Figure 1b). Despite these advancements [66,67], the number of ligand-modified uranyl complexes used in catalytic transformations remains scant [68,69,70,71,72,73,74]. Additionally, Schiff bases, a category of multidentate chelating ligands characterized by the presence of carbon–nitrogen double bonds, engage in coordination processes through the utilization of the lone pair of electrons situated on the nitrogen atom. This interaction results in the formation of a conjugated structure, which is inherently susceptible to π→π* transitions. Consequently, Schiff bases exhibit robust absorption capabilities within the ultraviolet spectral region, amplifying the light absorption potential of metal ions, particularly uranyl ions. This phenomenon, commonly referred to as the “antenna effect”, underscores the potential of Schiff base–uranyl compound synthesis to enhance the light absorption proficiency of uranyl ions in the ultraviolet spectrum, thereby augmenting photocatalytic efficiency. Here, we report a series of Salen-ligand-supported uranyl complexes (**1**–**6**) and assess their efficacy as photocatalysts for the activation and conversion of sp^3^ C-H bonds under visible light illumination. This study further highlights the potential of ligand-supported uranyl(VI) complexes in photocatalysis.

## 2. Results and Discussion

### 2.1. Synthesis and Structural Characterization 

**Scheme 1:**
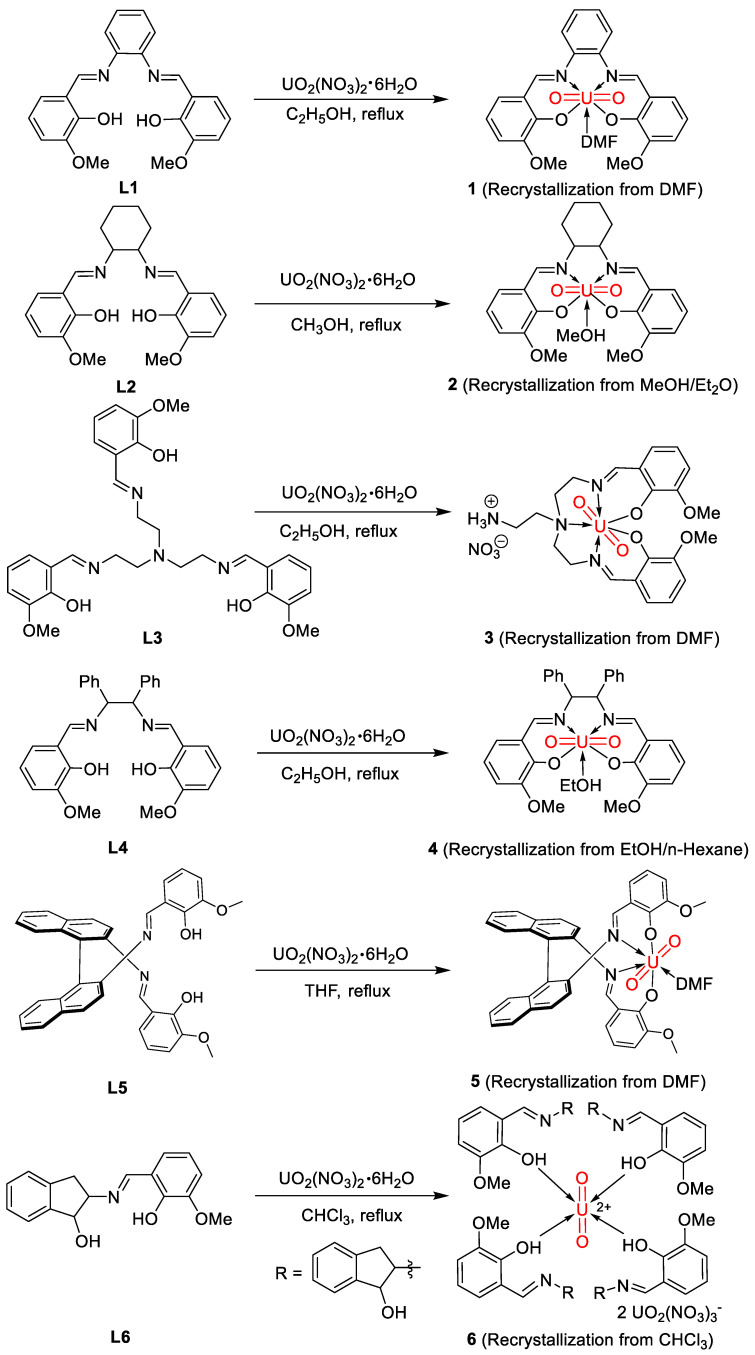
Synthesis of complexes **1**–**6**. The red color signifies the presence of uranyl ions.

Uranyl complexes were synthesized via the reactions of previously reported ligands **L1**–**L6** with one equivalent of UO_2_(NO_3_)_2_·6H_2_O under reflux conditions. Taking complex **1** as an example, a solution of **L1** in ethanol was combined with an ethanol solution of UO_2_(NO_3_)_2_·6H_2_O, followed by heating under reflux for 8 h. The reflux step is crucial for accelerating the reaction process, as it increases the reaction rate and shortens the crystallization time compared to non-reflux conditions. The color of the mixture changed from orange to red during this period. The resulting precipitate was filtered and washed sequentially with ethanol and ethyl ether. After about one week, red crystals of complex **1** were obtained in a 51% yield via the slow diffusion of N,N-dimethylformamide at room temperature. Complexes **2** to **6** were synthesized using similar procedures and were obtained with isolated yields ranging from 30% to 50% (Scheme 1). In the synthesis of the aforementioned uranyl complexes, we utilized not only ethanol but also other carefully selected solvents. For the synthesis of complex **2**, we found that using methanol as the solvent accelerated crystal precipitation and improved both the synthesis efficiency and the purity of the product. During the synthesis of complex **5**, we discovered that the biphenyl-diamine Schiff base ligand did not dissolve well in ethanol. By switching the solvent to tetrahydrofuran, we resolved this issue and facilitated the formation of the complex. For the synthesis of complex **6**, we initially used ethanol as the solvent and employed various recrystallization methods but were unable to obtain crystals. During an NMR experiment, we unexpectedly found that the compound dissolved well in chloroform. Subsequent experiments confirmed that using chloroform as the solvent enabled the successful crystallization of the target compound and significantly improved the yield of complex **6**. These complexes were comprehensively characterized by NMR spectroscopy, single-crystal X-ray diffraction, and elemental analysis (see Materials and Methods (Section 3) for details).

The ^1^H NMR spectra of these complexes, recorded in DMSO-d_6_ at room temperature, show only the Schiff base ligand peaks with notable chemical shift differences. The spectra reveal that all six complexes share structural similarities, each featuring two N=CH units and two OCH₃ groups. For instance, in the ^1^H NMR spectrum of complex **1**, the protons on the N=CH units and OCH₃ groups appear at 8.77 and 3.92 ppm, respectively, suggesting a symmetrical structure. For complex **2**, the peaks are at 9.37 ppm and 3.93 ppm; for complex **3**, at 9.68 ppm and 4.09 ppm; for complex **4**, at 9.35 ppm and 3.97 ppm; and for complex **5**, at 9.07 ppm and 3.62 ppm. Although there are slight variations in the N=CH and OCH₃ peaks, the overall structures are similar, with the primary differences arising from the chemical shifts of other parts of the ligand framework. For example, complex **1** contains a phenyl group, with peaks at 7.76–6.93 ppm; complex **2** contains a cyclohexyl group, with peaks at 4.02–1.16 ppm; complex **3** has a triamine framework, with peaks at 5.00–1.23 ppm; complex **4** contains two phenyl groups, with peaks at 7.61–6.25 ppm; and complex **5** has a binaphthyl group, with peaks at 8.25–6.75 ppm. Notably, the ^1^H NMR spectrum of complex **3** shows an additional peak at around 7.71 ppm, attributed to amino groups, consistent with its crystal structure. Interestingly, for complex **6**, the N=C-H proton is at 8.58 ppm, while aromatic hydrogens appear as multiple peaks between 7.32 and 6.85 ppm. A doublet at 4.82 ppm corresponds to the hydrogen on the carbon attached to the hydroxyl group in the penta-ring. OCH₃ hydrogens appear as a singlet at 3.69 ppm, and other penta-ring hydrogens show three sets of peaks between 3.92 and 3.16 ppm. The ^13^C NMR spectrum further confirms the successful formation of the product, with peaks at expected positions (see Materials and Methods (Section 3) for details).

**Figure 2:**
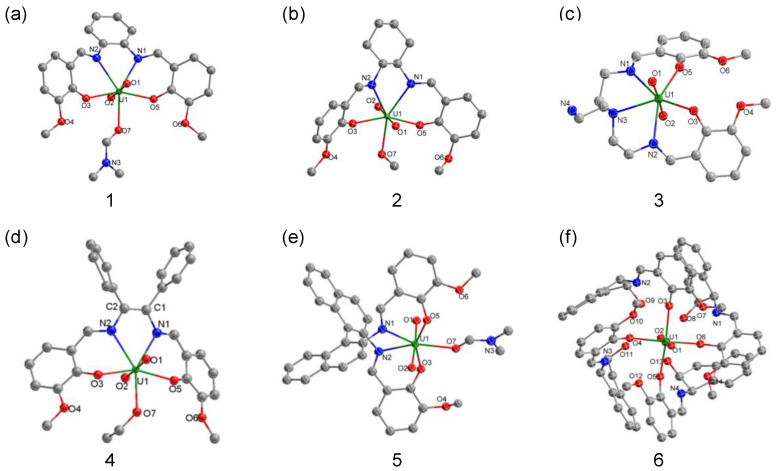
Molecular structures of **1** (**a**)**, 2** (**b**)**, 3** (**c**)**, 4** (**d**)**, 5** (**e**), and **6** (**f**) with thermal ellipsoids at the 50% probability level. The anions, solvent molecules, and hydrogen atoms are omitted for clarity. The color green is utilized to signify uranium elements, whereas the color red designates oxygen atoms, and the color blue designates nitrogen atoms.

The molecular structures of complexes **1** to **6** were determined by single-crystal X-ray diffraction analysis. As illustrated in Figure 2, the uranium centers in complexes **1** to **5** display a seven-coordinated pentagonal bipyramidal geometry, while the uranium center in complex **6** adopts a six-coordinated octahedral geometry. Given the structural similarities among complexes **1** to **5**, complex **1** was selected for detailed discussion. The bond lengths U1-O3 (2.246(2) Å) and U1-O5 (2.727(2) Å) in complex **1** are elongated compared to the shorter U1-O1 (1.793(3) Å) and U1-O2 (1.782(3) Å) bonds, aligning with established values for U-O bonds in uranyl(VI) complexes [75,76,77]. The shorter U1-O1 and U1-O2 bond lengths can be attributed to the orbital overlap between the uranium 6d and 5f orbitals with the p orbitals (or a combination of p and sp hybrid orbitals) of the axial oxygen atoms, fostering a linear arrangement. The U1-N1 (2.538(3) Å) and U1-N2 (2.535(3) Å) distances exceed typical U-N single bond lengths, affirming the coordinated nature of these N-U bonds. The U1-O7 length of 2.434(3) Å is notably longer than a standard U-O single bond, yet similar to the 2.491(9) Å reported for another uranyl(VI) Salen complex. In compounds **2**, **4** and **5**, the U1-O1 and U1-O2 bond lengths are 1.795(8) Å and 1.791(8) Å, 1.767(10) Å and 1.776(11) Å, and 1.783(4) Å and 1.782(4) Å, respectively. These values all fall within the range for U=O bonds in uranyl ions. The bond lengths for U1-N1 and U1-N2 are 2.542(10) Å and 2.575(9) Å, 2.498(13) Å and 2.552(12) Å, and 2.578(5) Å and 2.562(4) Å, respectively, all of which are longer than the sum of the covalent radii of U and N (2.41 Å), indicating that U and N form coordination bonds. The bond lengths for U1-O3 and U1-O5 are 2.250(8) Å and 2.248(8) Å, 2.289(13) Å and 2.272(9) Å, and 2.267(4) Å and 2.272(4) Å, all less than the sum of the covalent radii of U and O (2.33 Å), indicating that U and O3, O5 form covalent bonds. The bond length for U1-O7 ranges from 2.513(8) Å to 2.419(4) Å, falling within the range for coordination bonds.

Remarkably, complex **3** exhibited no solvent molecule coordination. Instead, the Schiff base ligand’s central nitrogen atom binds directly to the uranium center, yielding a U1-N3 bond of 2.665(2) Å, exceeding the covalent radius and indicative of a coordinate U-N bond [64]. Moreover, complex **3** features hydrolysis of one imine unit in ligand **L3**. The Schiff base ligand **L6** varies from the diamine ligands present in the other five complexes, resulting in the unique structure of complex **6**. The uranium center in complex **6** was coordinated with four ligands and encapsulates two UO_2_(NO_3_)_3_ anions. Across the series, with the exception of complex **6**, the uranium centers are bonded to two nitrogen atoms and two oxygen atoms from the ligands, flanked either by a solvent molecule or an extra nitrogen atom from the ligand in the equatorial plane. The selected bond lengths and bond angles for complexes **1**–**5** are shown in Table 1.

The ligand and complex spectra show characteristic bands that suggest the formation of the complexes [78,79], with tentative attributions summarized in Table 2. The infrared spectra of the complexes differ from those of their corresponding ligands, indicating the formation of new complexes. The C=N stretching vibration band of the *o*-phenylenediamine Schiff base ligand shifts from 1610 cm^−1^ to 1580 cm^−1^, suggesting the formation of a C=N-M bond system. The C-O vibration band of the ligand at 1248 cm^−1^ shifts lower by about 8 cm^−1^ in the metal complexes, indicating oxygen coordination to metal ions. The peak at 3100 cm^−1^ corresponds to the stretching vibration of methyl C-H, while the sharp peak at 1600 cm^−1^ aligns with the C=O stretching vibration, confirming the presence of DMF in the complex. The C=O bond in free DMF, typically at 1648 cm^−1^, shifts to a lower wavenumber in the complex, indicating DMF coordination with uranyl ions. For complex **2**, the C=N stretching vibration band shifts from 1630 cm^−1^ to 1620 cm^−1^ upon coordination, indicating nitrogen coordination with uranyl. The disappearance of the phenolic hydroxyl vibration band and the shift in the C-O vibration mode further confirm oxygen atom coordination. The infrared spectra also suggest the presence of water, hydroxyl groups, and nitrate ions in the complexes. In complex **3**, the C=N band shifts from 1645 cm^−1^ to 1620 cm^−1^, confirming coordination. A broad peak at 3500 cm^−1^ suggests the presence of water or hydroxyl groups. The shift in the C-O vibration band supports oxygen atom coordination. Additionally, the strong absorption at 1380 cm^−1^ and characteristic nitrate peaks at 1460 cm^−1^ and 1295 cm^−1^ indicate nitrate inclusion in the complex. In complex **4**, the C=N band shifts to 1608 cm^−1^, and a broad peak at 3400 cm^−1^ suggests the presence of water/alcohol molecules. For the binaphthyl diamine Schiff base ligand in complex **5**, the C=N band shifts to 1588 cm^−1^, and the disappearance of the phenolic hydroxyl vibration band confirms oxygen coordination. The C=O bond appears at 1620 cm^−1^, but in the complex, it shifts to a lower wavenumber, indicating that DMF coordinates with uranyl ions. In complex **6**, the C=N band shifts to 1618 cm^−1^. A broad peak at 3374 cm^−1^ in the hydroxyl vibration band indicates active hydrogen in both the benzopentane ring and phenolic hydroxyl groups. The UV-Vis spectra exhibited maximum absorption peaks for these Salen-ligand-supported uranyl complexes in the region of 310–320 nm. Additionally, an absorption peak around 410 nm is observed for the uranyl complexes, which is likely attributable to electron transfer from the Schiff base ligand to the metal. This absorption peak arises when the ligand contains higher energy lone pair electrons or when the metal possesses lower energy vacant orbitals. Such conditions can shift the absorption spectrum into the visible region, resulting in a noticeable color of the complex.

### 2.2. Photocatalytic Property 

Previous studies have demonstrated that uranyl(VI) can serve as an effective photocatalyst. In light of this, we examined the photocatalytic properties of newly synthesized complexes **1**–**6**. By employing dimethyl acetylenedicarboxylate (**7a**) and 1,3-dioxopentane (**8a**) as model substrates, we successfully achieved uranyl(VI)-photocatalyzed sp^3^ C-H bond activation and C-C bond formation. Under UV lamp irradiation, a reaction mixture comprising uranyl nitrate (5 mmol%), **7a**, and **8a** in acetone produced the target product with 18% efficiency (entry 1, Table 3). Through solvent optimization to acetonitrile, the yield was increased to 39%, but the isomerization ratio hardly changed (entry 2). Testing different uranyl complexes in this model reaction revealed that all exhibited higher efficiency and improved isomer ratios than uranyl nitrate, with complex **4** achieving the highest catalytic efficiency of 79% and a Z/E configuration ratio of 10:1 (entry 6). Our results underscored the critical role of light in catalytic performance, as no product formation occurred in the absence of light, even after 24 h (entry 9). Furthermore, the addition of TEMPO hindered the process, suggesting a potential radical-mediated mechanism (entry 10).

**Scheme 2:**
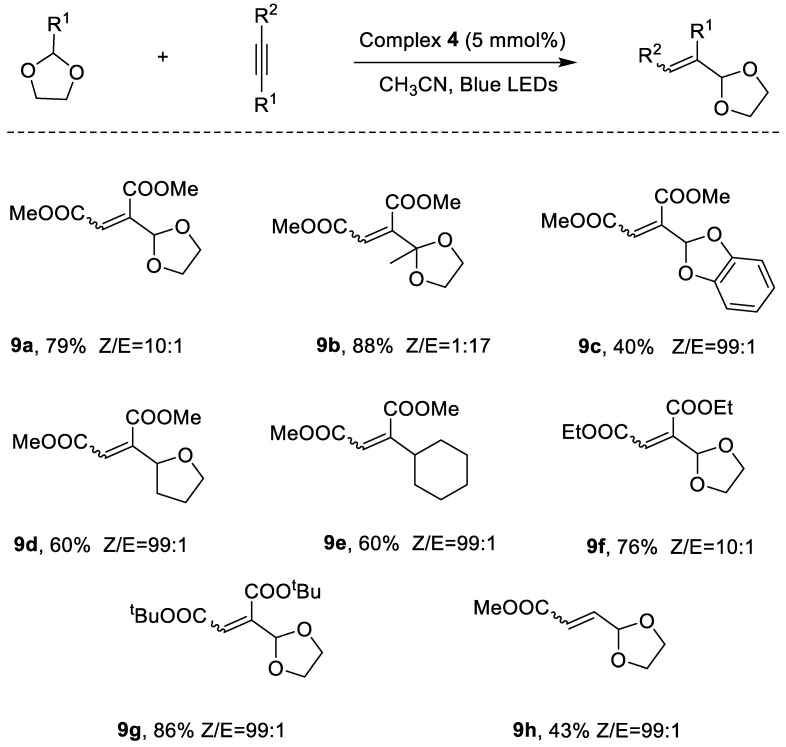
Scope for the photocatalyzed C−H activation by complex **4**.

Expanding upon these findings, we probed the reaction’s scope under optimized conditions. According to Scheme 2, modification of 1,3-dioxolane’s second position with a methyl group under milder conditions resulted in a product (**9b**) with an 88% yield and a Z/E isomer regioselectivity of 1:17. Changing the 1,3-dioxpentane to 1,3-benzodioxole led to a slight decrease in yield but predominantly produced the Z-isomer **9c**. Using tetrahydrofuran as the substrate under identical conditions yielded the Z-isomer (**9d**) with a moderate yield. Similarly, transforming cyclohexane under the same conditions produced the Z-isomer (**9e**) with a 60% yield.

We further investigated alkyne derivatives, using 1,3-dioxopentane as a partner (Scheme 2). Utilizing di-ethyl butynedioate as the acceptor yielded product **9f** with a 76% yield and a Z/E configuration ratio of 10:1. The yield for **9g** surged to 86% when using di-tert-butyl butynedioate as the acceptor, exclusively yielding the Z-configuration. Conversely, employing methyl propiolate as the acceptor led to a mere 43% yield for **9h**, likely due to the alkyne’s reduced electrophilicity.

**Figure 3:**
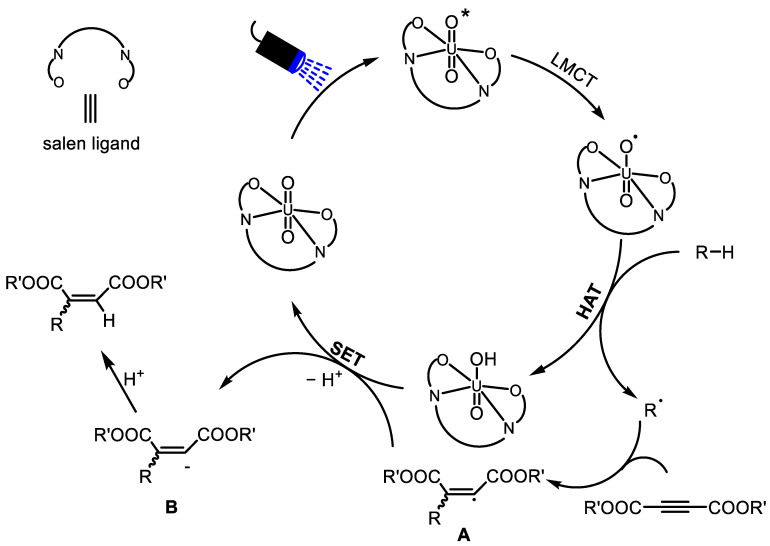
Proposed mechanism.

A proposed reaction mechanism is depicted in Figure 3. This reaction begins with the excitation of the uranyl cation under visible-light irradiation, leading to the formation of an excited uranyl species. This species can abstract a hydrogen atom from the substrate molecule, resulting in the creation of a C-centered radical. This radical is subsequently intercepted by electron-deficient alkynes, forming radical intermediate **A**. This intermediate then undergoes a single-electron transfer process to convert into species **B** and regenerate the uranyl cations. The intermediate **B** reacts with H^+^ to yield the final products, thus completing the catalytic cycle.

## 3. Materials and Methods

### Materials and Instruments

All reagents were commercially available and used without further purification. Nuclear magnetic resonance (NMR) spectroscopy was performed using a Bruker AVIII-400 spectrometer (Ettlingen, Germany) (^1^H 400 MHz; ^13^C{^1^H} 101 MHz) at room temperature. The ^1^H NMR chemical shifts (δ) are referenced to tetramethylsilane. The absolute values of coupling constants are reported in hertz (Hz). Multiplicities are denoted as singlet (s), doublet (d), triplet (t), multiplet (m), and broad (br). Elemental analyses (C, H, N) were carried out using a Vario MICRO cube elemental analyzer at the Center for Modern Analysis, Nanjing University. Fourier transform infrared (FT-IR) spectra were recorded using a Bruker-VERTEX80V instrument. UV-visible-NIR absorption spectra were obtained using a Shimadzu Corporation-UV3600 spectrophotometer. The ligands **L1** through **L6** were synthesized according to previously reported procedures [80,81,82,83,84].

Single-crystal X-ray diffraction data [85] for complexes **1**, **2**, **3**, **4**, **5**, and **6** were collected using a BRUKER D8 VENTURE PHOTON II detector with a radiation source of Mo(Kα) (0.71073 Å) or Ga(Kα) (1.34139 Å). All structures were solved by Patterson methods and refined on F2 using full-matrix least-squares methods with the SHELXTL-2014 program package. All non-hydrogen atoms were refined on F2 by full-matrix least-squares procedures with the use of anisotropic displacement parameters. Hydrogen atoms were introduced at their geometric positions and refined as riding atoms. Details regarding the data collection and refinement for these complexes were given in Tables S1 and S2 in the Supplementary Materials.

Synthesis of complex **1**: A solution of **L1** (18.0 mg, 0.5 mmol, 1 equiv.) in ethanol (15 mL) was combined with an ethanol solution of uranyl nitrate (25.0 mg, 0.5 mmol, 1 equiv.), followed by heating under reflux for 8 h. The color of the mixture changed from orange to red during this period. The resulting precipitate was filtered and washed sequentially with ethanol and ethyl ether. After about one week, red strip-shaped crystals of compound **1** (16.0 mg, 51% yield) were obtained via the slow diffusion of N,N-dimethylformamide at room temperature. ^1^H NMR (400 MHz, DMSO-d_6_, 298 K): δ(ppm): 8.77 (s, 2H, HC=N), 7.37–7.31 (m, 2H, Ar-H), 7.54–7.17 (m, 2H, Ar-H), 7.40 (dd, *J* = 8.0 Hz, 1.4 Hz, 2H, Ar-H), 7.25 (dd, *J* = 7.8 Hz, 1.6 Hz, 2H, Ar-H), 6.63 (t, 2H, *J* = 7.8 Hz, Ar-H), 3.92 (s, 6H, OCH_3_). ^13^C{^1^H} NMR (100 MHz, DMSO-d_6_, 298 K): δ(ppm): 166.4, 160.9, 151.0, 146.7, 128.6, 127.2, 124.0, 120.2, 117.3, 115.8, 56.0. Anal. Calcd. For (C_22_H_18_N_2_O_6_U): C, 41.00; H, 2.82; N, 4.35. Found: C, 41.03; H, 3.00; N, 4.38.

Synthesis of complex **2**: A solution of **L2** (19.1 mg, 0.5 mmol, 1 equiv.) in methanol (15 mL) was added to a methanol solution of uranyl nitrate (25.0 mg, 0.5 mmol, 1 equiv.), and the mixture was refluxed for 10 h. Over this time, the color transitioned from yellow to red. Red strip-shaped crystals of compound **2** (18.0 mg, 53% yield) formed after approximately one week through the slow diffusion of ethyl ether into the filtrate at room temperature. ^1^H NMR (400 MHz, DMSO-d_6_, 298 K): δ(ppm): 9.37 (s, 2H, HC=N), 7.21 (dd, *J* = 7.9 Hz, 1.6 Hz, 2H, Ar-H), 7.16 (dd, *J* = 7.9 Hz, 1.6 Hz, 2H, Ar-H), 6.55 (t, *J* = 7.8 Hz, 2H, Ar-H), 4.02–3.98(m, 2H, N-Cy-H), 3.93 (s, 6H, OCH_3_), 2.46–2.04 (m, 2H, Cy-H), 2.02–1.95(m, 2H, Cy-H), 1.90–1.80 (m, 2H, Cy-H), 1.54–1.45 (m, 2H, Cy-H). ^13^C{^1^H} NMR (100 MHz, DMSO-d_6_, 298 K): δ(ppm): 164.6, 156.0, 144.8, 131.2, 129.0, 121.6, 120.5, 75.8, 61.2, 36.8, 29.9. Anal. Calcd. For(C_24_H_32_N_2_O_8_U): C, 40.42; H, 4.14; N, 4.10. Found: C, 40.66; H,4.30; N, 4.16.

Synthesis of complex **3**: A solution of **L3** (27.5 mg, 0.5 mmol, 1 equiv.) in ethanol (15 mL) was combined with an ethanol solution of uranyl nitrate (25.0 mg, 0.5 mmol, 1 equiv.) and heated under reflux for 8 h. The reaction mixture changed color from yellow to orange. After filtration and sequential washing with ethanol and n-hexane, red strip-shaped crystals of compound **3** (19.0 mg, 51% yield) were collected after about a week via the slow diffusion of N,N-dimethylformamide at room temperature. ^1^H NMR (400 MHz, DMSO-d_6_, 298 K): δ(ppm): 9.68 (s, 2H, HC=N), 7.71 (s, 3H, NH_3_^+^), 7.25 (d, *J* = 7.8 Hz, 4H, Ar-H), 6.63 (t, *J* = 7.8 Hz, 2H, Ar-H), 4.97 (t, *J* = 14.2 Hz, 2H, NCH_2_), 4.63 (dd, *J* = 15.8 Hz, 4.4Hz, 2H, NCH_2_-Ar), 4.09 (s, 6H, OCH_3_), 3.98 (dd, *J* = 13.1 Hz, 3.5 Hz, 2H, NCH_2_), 3.85–3.70 (m, 2H, NCH_2_-Ar), 3.68 (td, 2H, *J* = 13.4 Hz, 4.7 Hz, NCH_2_-Ar), 3.42–3.68 (m, 2H, NCH_2_). ^13^C{^1^H} NMR (100 MHz, DMSO-d_6_, 298 K): δ(ppm): 170.7, 159.7, 150.9, 126.6, 123.7, 118.7, 116.4, 57.4, 57.0, 54.7, 45.1, 31.4. Anal. Calcd. For(C_22_H_29_N_5_O_9_U): C, 35.44; H, 3.92; N, 9.39. Found: C, 35.60; H, 4.02; N, 9.59.

Synthesis of complex **4**: A solution of **L4** (24.0 mg, 0.5 mmol, 1 equiv.) in ethanol (20 mL) was mixed with an ethanol solution of uranyl nitrate (25.0 mg, 0.5 mmol, 1 equiv.), and refluxed for 8 h. The reaction mixture’s color changed from yellow to red during this period. Red strip-shaped crystals of compound **4** (18.0 mg, 53% yield) were obtained after approximately three days through the slow diffusion of n-hexane into the filtrate at room temperature. ^1^H NMR (400 MHz, DMSO-d_6_, 298 K): δ(ppm): 9.35 (s, 2H, HC=N), 7.64–7.56 (m, 4H, Ar-H), 7.33–6.98 (m, 10H, Ph-H), 6.57 (t, *J* = 7.8 Hz, 2H, Ar-H), 6.25 (s, 2H, NHC=CHN), 3.97 (s, 6H, OCH_3_). ^13^C{^1^H} NMR (100 MHz, DMSO-d_6_, 298 K): δ(ppm): 171.1, 160.3, 150.8, 141.5, 128.1, 127.3, 127.1, 126.2, 123.0, 116.7, 115.5, 79.6, 56.0. Anal. Calcd. For(C_31_H_28_N_2_O_7_U): C,47.82; H, 3.62; N, 3.60. Found: C, 47.89; H, 3.60; N, 3.70.

Synthesis of complex **5**: **L5** (28.0 mg, 0.5 mmol, 1 equiv.) was dissolved in tetrahydrofuran (15 mL) and added to a tetrahydrofuran solution of uranyl nitrate (25.0 mg, 0.5 mmol, 1 equiv.), followed by refluxing for 8 h. The mixture’s color changed from orange to red. The precipitate was filtered and washed with tetrahydrofuran and ethyl ether. Red strip-shaped crystals of compound **5** (17.0 mg, 42% yield) were obtained after about two weeks via the slow diffusion of N,N-dimethylformamide at room temperature. ^1^H NMR (400 MHz, DMSO-d_6_, 298 K): δ(ppm): ^1^H NMR (400 MHz, DMSO-d_6_, 298 K): δ(ppm): 9.07 (s, 2H, HC=N), 8.24 (d, *J* = 8.4 Hz, 2H, Binaphthal-H), 8.08 (d, *J* = 8.2 Hz, 2H, Binaphthal-H), 8.00 (d, *J* = 9.0 Hz, 2H, Binaphthal-H), 7.49 (ddd, *J* = 8.1 Hz, 6.8 Hz, 1.2 Hz, 2H, Binaphthal-H), 7.31 (ddd, *J* = 8.3 Hz, 6.9 Hz, 1.3 Hz, 2H, Binaphthal-H), 7.07 (dd, *J* = 7.9 Hz, 1.5 Hz, 2H, Binaphthal-H), 7.01 (dd, *J* = 8.5 Hz, 1.1 Hz, 2H, Ar-H), 6.94 (dd, *J* = 8.0 Hz,1.4 Hz, 2H, Ar-H), 6.77 (t, *J* = 7.9 Hz, 2H, Ar-H), 3.62 (s, 6H, OCH_3_). ^13^C{^1^H} NMR (100 MHz, DMSO-d_6_, 298 K): δ(ppm): 163.0, 150.0, 147.5, 142.9, 132.5, 132.2, 129.8, 129.3, 128.3, 127.0, 125.9, 125.6, 123.9, 118.8, 118.3, 117.2, 115.0, 55.3. Anal. Calcd. For(C_37_H_28_N_2_O_6_U): C,53.24; H, 3.38; N, 3.36. Found: C, 53.66; H, 3.40; N, 3.40.

Synthesis of complex **6**: **L6** (14.0 mg, 0.5 mmol, 1 equiv.) in chloroform (20 mL) was added to a chloroform solution of uranyl nitrate (25.0 mg, 0.5 mmol, 1 equiv.), which was then refluxed for 12 h. The reaction mixture changed from yellow to red in color. Red strip-shaped crystals of compound **6** (23.2 mg, 36% yield) formed after approximately three weeks through the slow diffusion of chloroform at room temperature. ^1^H NMR (400 MHz, DMSO-d_6_, 298 K): δ(ppm): 13.39 (s, 4H, OH), 8.58 (s, 4H, CH=N), 7.32–7.26 (m, 8H, Indan-H), 7.23–7.16(m, 8H, Indan-H), 6.97–6.94 (m, 8H, Ar-H), 6.85 (t, *J* = 8.3 Hz, 4H, Ar-H), 4.82 (d, *J* = 5.3 Hz, 4H, CH-O), 4.07 (s, 4H, OH), 3.92–3.88 (m, 4H, Indan-H), 3.69 (s, 12H, OCH_3_), 3.28 (dd, *J* = 15.9 Hz, 5.9 Hz, 4H, Indan-H), 3.16 (dd, *J* = 15.9 Hz, 5.3 Hz, 4H, Indan-H) ^13^C{^1^H} NMR (100 MHz, DMSO-d_6_, 298 K): δ(ppm): 167.2, 161.6, 157.2, 152.2, 141.1, 140.9, 129.8, 127.5, 125.9, 125.3, 123.8, 118.7, 115.0, 75.7, 75.5, 56.6, 40.0. Anal. Calcd.For(C_70_H_72_N_4_O_13_U): C,59.40; H, 5.13; N, 3.96. Found: C, 59.84; H, 5.16; N, 4.29.

General procedure for U(VI) photocatalytic reaction: To a sealed vial equipped with a magnetic stir bar, the uranyl(VI) complex (5 mol%, 3.2 mg), electron-deficient alkyne (1.0 equiv.), corresponding substrate (1.0 equiv.), and anhydrous acetonitrile (MeCN; 0.2 M) were added. The vial was sealed with a screw cap and subjected to irradiation under blue LEDs (3W × 4) at room temperature for 24 h. The desired products were isolated post-reaction via purification by silica gel column chromatography using petroleum ether/ethyl acetate (20:1).

**9a** was prepared with 1,3-dioxolane (0.2 mmol), yielding a colorless oil (17.0 mg, 79%). ^1^H NMR (400 MHz, CDCl_3_) δ 6.78 (dd, *J* = 15.8, 4.6 Hz, 1H), 5.70 (dd, *J* = 15.7, 1.1 Hz, 1H), 4.00–3.99 (m, 2H), 3.98–3.96 (m, 2H), 3.78 (s, 3H), 3.75 (s, 3H). ^13^C{^1^H} NMR (100 MHz, CDCl_3_): δ(ppm): 166.1, 165.2, 144.6, 122.7, 100.7, 65.2, 52.7, 52.6. HRMS (ESI) *m/z*: [M + Na]^+^ Calcd for C_10_H_14_O_6_ 239.0526; Found 239.0519.

**9b** was obtained using 2-methyl-1,3-dioxolane (0.2 mmol), yielding a colorless oil (20.0 mg, 60%). ^1^H NMR (400 MHz, CDCl_3_) δ 6.66 (s, 1H), 3.95–3.93 (m, 2H), 3.92–3.90 (m, 2H), 3.79 (s, 3H), 3.78 (s, 3H), 1.77 (s, 3H). ^13^C{^1^H} NMR (100 MHz, CDCl_3_): δ(ppm): 166.4, 165.8, 140.6, 129.0, 119.7, 65.0, 52.5, 52.2, 25.3. HRMS (ESI) *m/z*: [M + Na]^+^ Calcd for C_12_H_18_O_4_ 253.0682; Found 253.0676.

**9c** was synthesized with 1,3-benzodioxole (0.2 mmol), yielding a yellow oil (10.0 mg, 40%). ^1^H NMR (400 MHz, CDCl_3_) δ 6.85 (s, 4H), 6.79 (d, *J* =1.1 Hz, 1H), 6.50 (d, *J* =1.0 Hz, 1H), 3.86 (s, 3H), 3.78 (s, 3H). ^13^C{^1^H} NMR (100 MHz, CDCl_3_): δ(ppm): 165.3, 164.7, 146.4, 139.6, 126.3, 122.3, 109.1, 105.7, 52.9, 52.55. HRMS (ESI) *m/z*: [M + Na]^+^ Calcd for C_13_H_12_O_6_ 287.0526; Found 287.0503.

**9d** was generated from tetrahydrofuran (0.2 mmol), yielding a colorless oil (12.2 mg, 60%). ^1^H NMR (400 MHz, CDCl_3_) δ 6.10 (d, *J* =1.7 Hz, 1H), 4.66 (t, *J* = 5.7 Hz, 1H), 3.96–3.91 (m, 2H), 3.83 (s, 3H), 3.73 (s, 3H), 2.18–2.09 (m, 2H), 1.97–1.88 (m, 2H). ^13^C{^1^H} NMR (100 MHz, CDCl_3_): δ(ppm): 167.7, 165.7, 150.9, 118.4, 78.0, 69.1, 52.6, 52.0, 31.2, 25.4. HRMS (ESI) *m/z*: [M + Na]^+^ Calcd for C_10_H_14_O_5_ 237.0733; Found 237.0729.

**9e** was produced with cyclohexane (0.2 mmol), yielding a colorless oil (13.1 mg, 88%). ^1^H NMR (400 MHz, CDCl_3_) δ 5.76 (d, *J* =1.4 Hz, Hz, 1H), 3.82 (s, 3H), 3.70 (s, 3H), 2.31–2.25 (m, 1H), 1.85–1.77 (m, 4H), 1.71–1.66 (m, 1H), 1.31–1.10 (m, 5H) ^13^C{^1^H} NMR (100 MHz, CDCl_3_): δ(ppm): 168.8, 165.9, 156.8, 117.4, 52.6, 52.2, 42.8, 31.5, 26.4, 26.1. HRMS (ESI) *m/z*: [M + Na]^+^ Calcd for C_12_H_18_O_4_ 249.1097; Found 249.1090.

**9f** was derived from diethyl acetylenedicarboxylate (17.0 mg, 0.1 mmol), yielding a colorless oil (18.0 mg, 86%). ^1^H NMR (400 MHz, CDCl_3_) δ 6.24 (d, *J* =1.1 Hz, 1H), 5.71 (d, *J* =1.2 Hz, 1H), 4.31 (q, *J* = 7.4 Hz, 2H), 4.21 (q, *J* = 7.1 Hz, 2H), 4.01–3.99 (m, 2H), 3.98–3.96 (m, 2H), 1.33 (t, *J* =7.1 Hz, 3H), 1.28 (t, *J* =7.1 Hz, 3H). ^13^C{^1^H} NMR (100 MHz, CDCl_3_): δ(ppm): 165.6, 164.8, 144.4, 123.0, 100.8, 65.2, 61.7, 61.3, 14.1. HRMS (ESI) *m/z*: [M + Na]^+^ Calcd for C_11_H_16_O_6_ 267.0839; Found 267.0832.

**9g** was prepared with di-tert-butyl acetylenedicarboxylate (23.0 mg, 0.1 mmol), yielding a colorless oil (25.2 mg, 76%). ^1^H NMR δ(400 MHz, CDCl_3_) 6.09 (t, *J* = 1.0 Hz, 1H), 5.66 (d, *J* = 1.0 Hz, 1H), 4.01–3.98(m, 2H), 3.97–3.95 (m, 2H), 1.53 (s, 9H), 1.48 (s, 9H). ^13^C{^1^H} NMR (100 MHz, CDCl_3_): δ(ppm): 164.6, 164.0, 143.7, 123.6, 101.1, 82.5, 81.5, 64.9, 28.2. HRMS (ESI) *m/z*: [M + Na]^+^ Calcd for C_15_H_24_O_6_ 323.1465; Found 323.1458.

**9h** was synthesized using methyl propiolate (9.0 mg, 0.1 mmol), yielding a colorless oil (6.5 mg, 43%). ^1^H NMR (400 MHz, CDCl_3_) δ 6.78 (dd, *J* = 15.8 Hz, 4.6 Hz, 1H), 6.16 (dd, *J* = 15.7 Hz, 1.1 Hz, 1H), 5.46 (dd, *J* = 4.7 Hz, 1.1 Hz, 1H), 4.01–3.98 (m, 2H), 3.97–3.92 (m, 2H), 3.76 (s, 3H). ^13^C{^1^H} NMR (100 MHz, CDCl_3_): δ(ppm): 163.1, 142.4, 124.4, 101.6, 65.5, 52.2. HRMS (ESI) *m/z*: [M + Na]^+^ Calcd for C_7_H_10_O_4_ 181.0471; Found 181.0459.

## 4. Conclusions

In summary, the successful synthesis of uranyl complexes through the reaction of ligands **L1**–**L6** with UO₂(NO₃)₂·6H₂O under reflux conditions yielded products **1**–**6** with yields ranging from 30% to 50%. These complexes were characterized using NMR spectroscopy, single-crystal X-ray diffraction, and elemental analysis. X-ray diffraction analysis revealed that complexes **1**–**5** adopted a seven-coordinated pentagonal bipyramidal geometry, whereas complex **6** exhibited a distinct six-coordinated octahedral geometry. Notably, complex **3** featured direct nitrogen bonding to uranium without solvent coordination, and one imine unit underwent hydrolysis. In contrast, complex **6**, with four ligands coordinated to a single uranyl ion, is likely indicative of the monodentate nature of its ligand. These complexes exhibit effective photocatalytic capabilities, especially in the activation of sp^3^ C-H bonds and the formation of C-C bonds. Newly synthesized uranyl complexes **1**–**6** were employed, emphasizing the activation of sp^3^ C-H bonds and the formation of C-C bonds, utilizing dimethyl acetylenedicarboxylate (**7a**) and 1,3-dioxopentane (**8a**) as model substrates. Notably, complex **4** emerged as the most efficient among the tested complexes, exhibiting a yield of 79% and an isomer ratio of Z/E = 10:1. Under optimized conditions, the reaction scope was broadened, revealing high yields and regioselectivity for a diverse range of substrates, encompassing modified 1,3-dioxolanes, 1,3-benzodioxole, tetrahydrofuran, and cyclohexane. Extending previous research, the study also delved into the reaction of 1,3-dioxopentane with various alkyne derivatives. Specifically, the use of diethyl butynedioate yielded 76% of product **9f** with a Z/E ratio of 10:1, whereas di-tert-butyl butynedioate achieved an even higher yield of 86%, exclusively producing the Z-configuration product **9g**. Conversely, methyl propiolate, owing to its diminished electrophilicity, resulted in a lower yield of 43% for **9h**. The proposed reaction mechanism involves the visible-light excitation of the uranyl cation, resulting in the abstraction of a hydrogen atom from the substrate and the generation of a carbon-centered radical. This radical is then intercepted by electron-deficient alkynes, enabling the formation of an intermediate. This characteristic light absorption property of the uranyl complexes can potentially augment the optical responsiveness of the molecule, rendering them highly suitable for applications in optical sensors and photocatalysis.

## Figures and Tables

**Table 1:** Selected bond lengths (Å) and bond angles (^o^) for complexes **1**–**5**.

	1	2	3	4	5
U1-O1	1.793(3)	1.795(8)	1.776(3)	1.767(10)	1.783(4)
U1-O2	1.782(3)	1.791(8)	1.765(3)	1.776(11)	1.782(4)
U1-O3	2.246(3)	2.250(8)	2.222(2)	2.289(10)	2.267(4)
U1-O5	2.272(3)	2.248(8)	2.261(3)	2.272(9)	2.272(4)
U1-O7	2.434(3)	2.513(8)	-	2.44(4)	2.419(4)
U1-N1	2.538(3)	2.542(10)	2.571(3)	2.498(13)	2.578(5)
U1-N2	2.535(3)	2.575(9)	2.544(3)	2.552(12)	2.562(4)
O1-U1-O2	175.34(12)	178.3(4)	176.01(14)	176.8(5)	176.10(19)
O3-U1-O5	154.44(9)	154.9(3)	85.28(11)	156.1(4)	153.51(16)
N1-U1-N2	63.89(9)	64.7(3)	131.17(12)	63.2(4)	70.09(14)

**Table 2:** Selected IR data of each ligand and its complexes (cm^−1^).

	ν(C=N)	ν(O-H)	ν(C=O)_DMF_	ν(C-O)	σ(U-O)	ν(NO_3_^−^)
**L1**	1612	3420		1248		
**1**	1580		1620	1240	497	
**L2**	1630	3500		1270		
**2**	1620			1243	494	
**L3**	1645	3500		1276		
**3**	1625			1245	500	1460, 1380, 1295
**L4**	1630	3400		1255		
**4**	1608			1246	499	
**L5**	1603	3400		1223		
**5**	1588		1620	1250	487	
**L6**	1635	3500		1282		
**6**	1618	3374		1251	473	

**Table 3:** Control experiments for the uranyl(VI) photocatalyzed C−H activation ^a^.

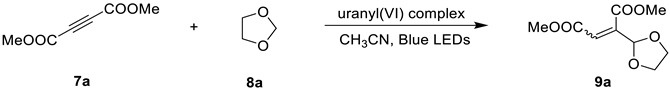				
Entry	Catalyst (5 mol%)	Solvent	Yield (%)	Ratio of Z/E
1	UO_2_(NO_3_)_2_·6H_2_O	(CH_3_)_2_CO	18	2:1
2	UO_2_(NO_3_)_2_·6H_2_O	CH_3_CN	39	2:1
3	Complex **1**	CH_3_CN	41	6:1
4	Complex **2**	CH_3_CN	59	6:1
5	Complex **3**	CH_3_CN	61	5:1
**6**	**Complex 4**	**CH_3_CN**	**79**	**10:1**
7	Complex **5**	CH_3_CN	67	10:1
8	Complex **6**	CH_3_CN	51	4:1
9 ^b^	Complex **4**	CH_3_CN	N.D	-
10 ^c^	Complex **4**	CH_3_CN	N.D	-

^a^ **7a** (0.1 mmol), **8a** (0.2 mmol), MeCN 0.5 mL, 5 mol% of uranyl(VI) complex, room temperature, under the air atmosphere conditions, blue LEDs, 24 h. ^b^ no light. ^c^ add TEMPO.

## Data Availability

The original contributions presented in the study are included in the article/Supplementary Material, further inquiries can be directed to the corresponding author.

## Supplementary Materials

The following supporting information can be downloaded at: https://www.mdpi.com/article/10.3390/molecules29174077/s1, Figures S1–S28: NMR spectra; Figures S29–S34: UV-visible absorption spectra; Figures S35–S40: FT-IR spectra; Figures S41–S46: Solid-state structure; Tables S1–S2: Crystal data and structural refinement; Tables S3–S8: Selected bond distances and angles for all complexes **1**–**6**.
