# Supplementary material for: Synthesis and Photocatalytic sp3 C-H Bond Functionalization of Salen-Ligand-Supported Uranyl(VI) Complexes
Source: Molecules. 2024 Aug 28;29(17):4077. doi: 10.3390/molecules29174077 (PMC11397425; doi:10.3390/molecules29174077)
Supplement: Supplementary file 1 [file molecules-29-04077-s001.zip › molecules-3144940-supplementary.pdf]

Supporting Information for

Synthesis and Photocatalytic sp^3 C-H Bond Functionalization of Salen-Ligand-Supported Uranyl(VI) Complexes

Jialu He [1], Xingxing Gong [1], Yafei Li [1], Qianyi Zhao [2] and Congqing Zhu [1,*]

[1] State Key Laboratory of Coordination Chemistry, Jiangsu Key Laboratory of Advanced Organic Materials, School of Chemistry and Chemical Engineering, Nanjing University, Nanjing 210023, China; jialuhe1228@163.com (J.H.); dg21240032@smail.nju.edu.cn (X.G.); 17865187280@163.com (Y.L.)

[2] School of Chemistry and Chemical Engineering, Henan Normal University, Xinxiang 453007, China; qyzhao@htu.edu.cn

* Correspondence: zcq@nju.edu.cn

Contents

1. Supporting Figures……………………………………………………S2

2. X-ray Crystallographic Analysis……………………………………S22

1. Supporting Figures

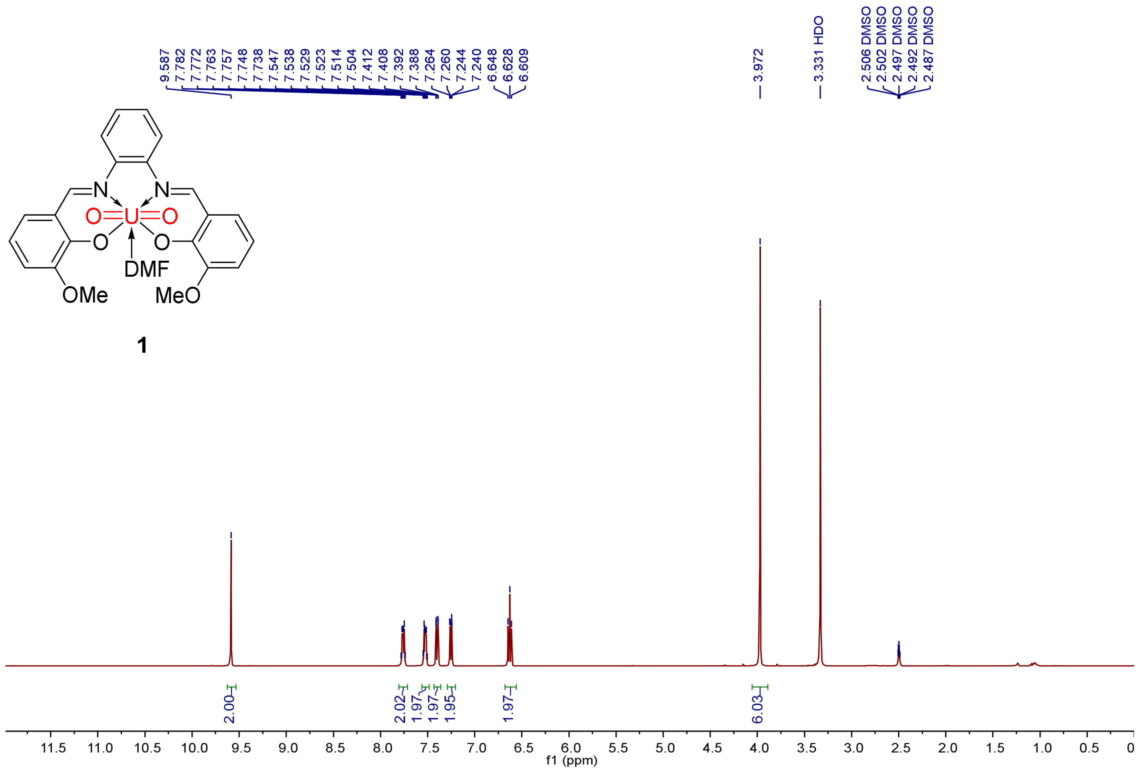

Figure S1. ^{1}H NMR spectrum of compound **1** (DMSO-d$_6$, 400 MHz)

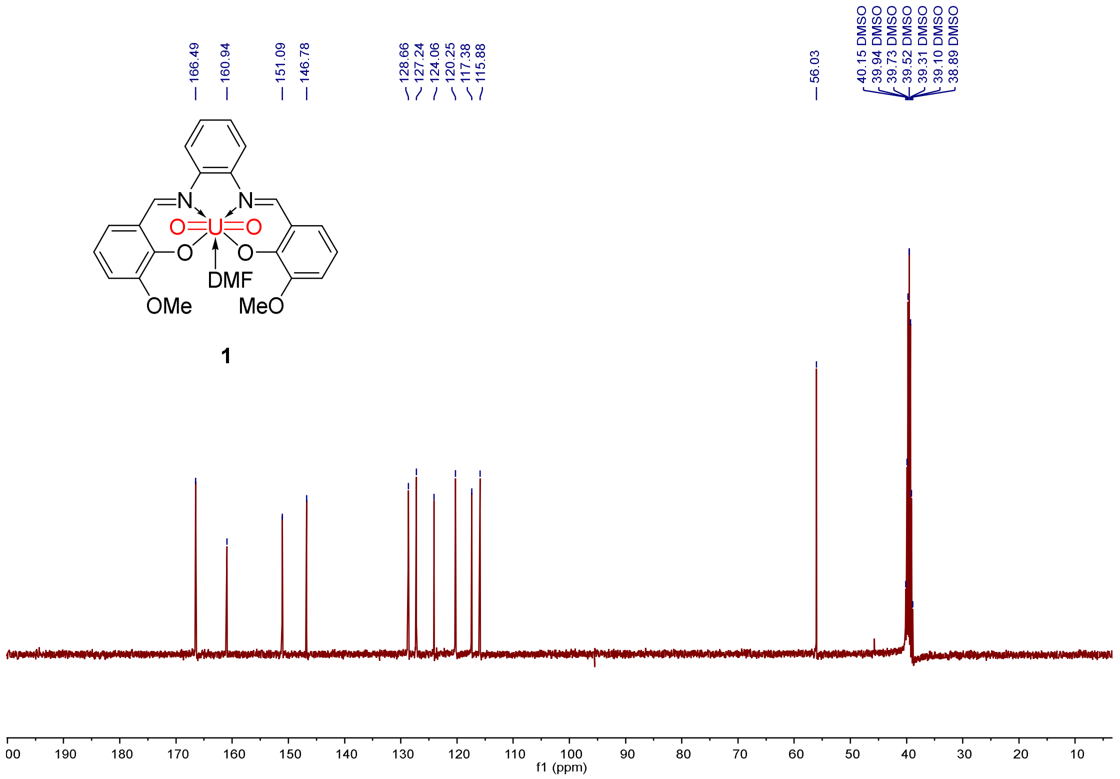

Figure S2. $^{13}C\{^{1}H\}$ NMR spectrum of compound **1** (DMSO-d$_6$, 100 MHz)

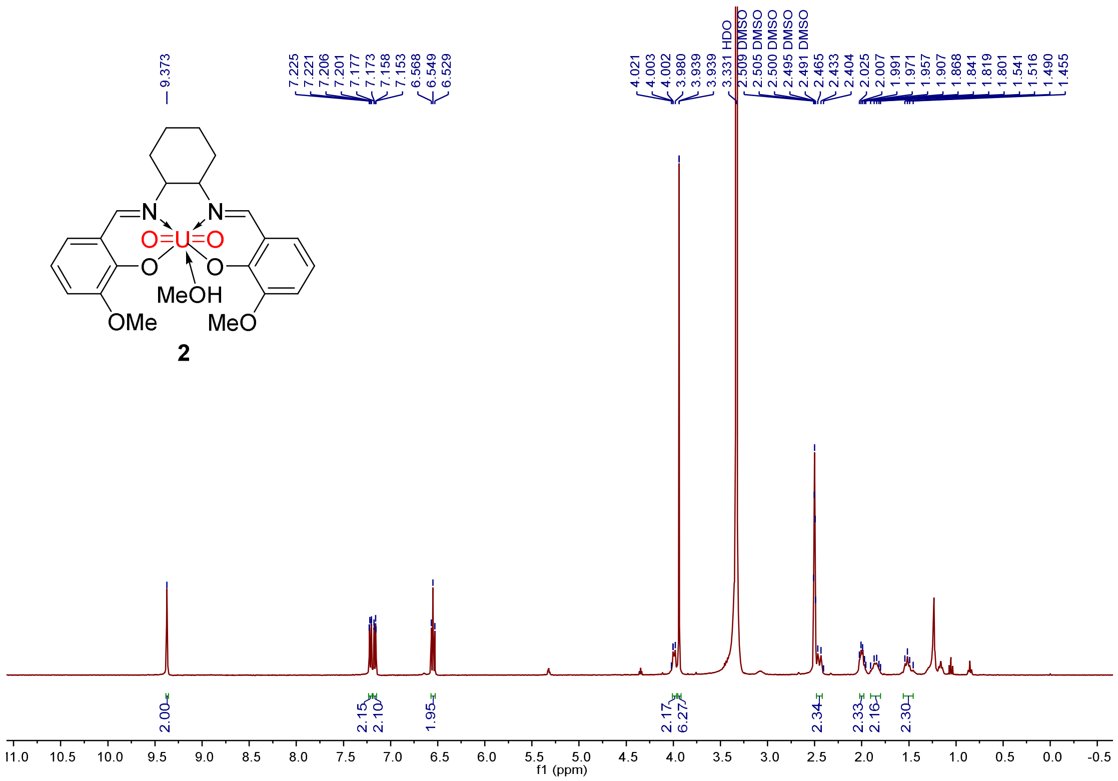

Figure S3. ^{1}H NMR spectrum of compound **2** (DMSO-d_6, 400 MHz)

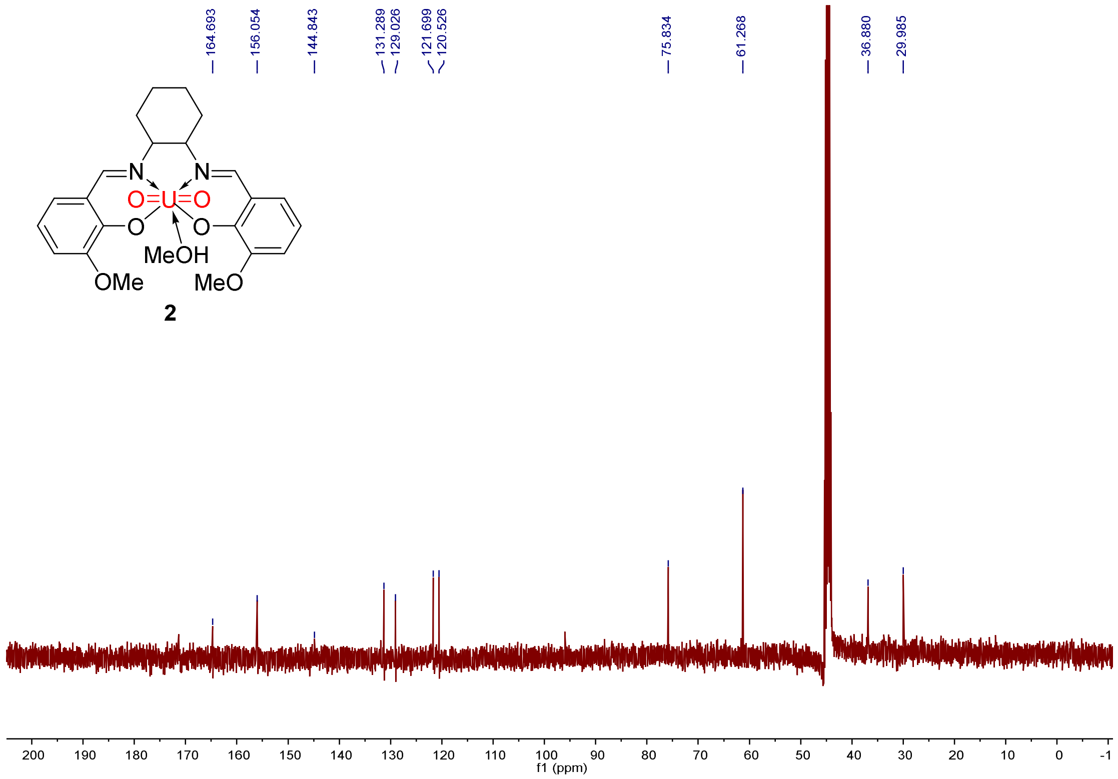

Figure S4. $^{13}C\{^{1}H\}$ NMR spectrum of compound **2** (DMSO-d_6, 100 MHz)

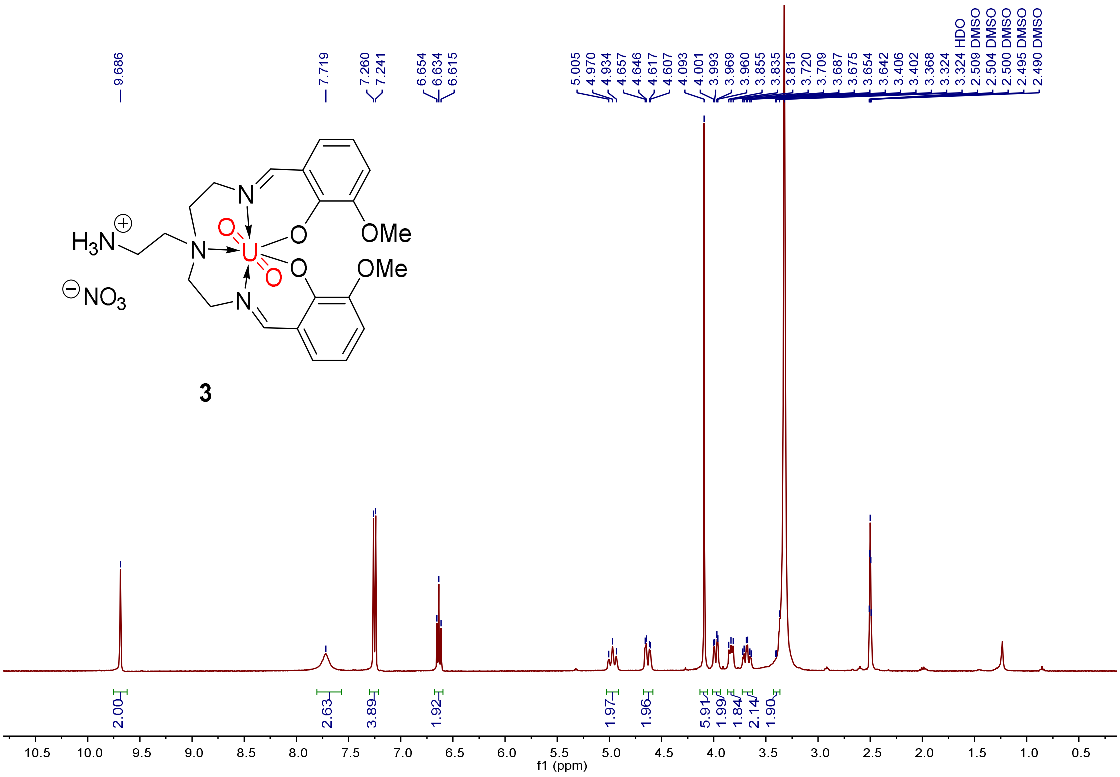

Figure S5. ^{1}H NMR spectrum of compound **3** (DMSO-d$_6$, 400 MHz)

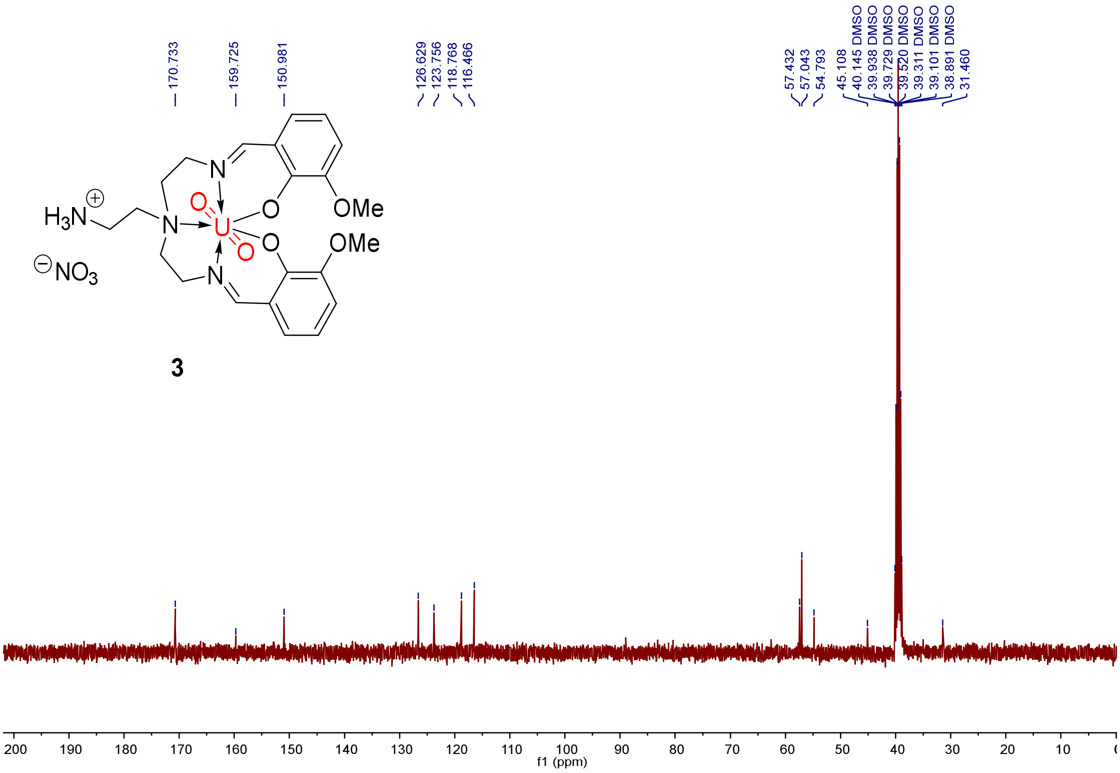

Figure S6. $^{13}C\{^{1}H\}$ NMR spectrum of compound **3** (DMSO-d$_6$, 100 MHz)

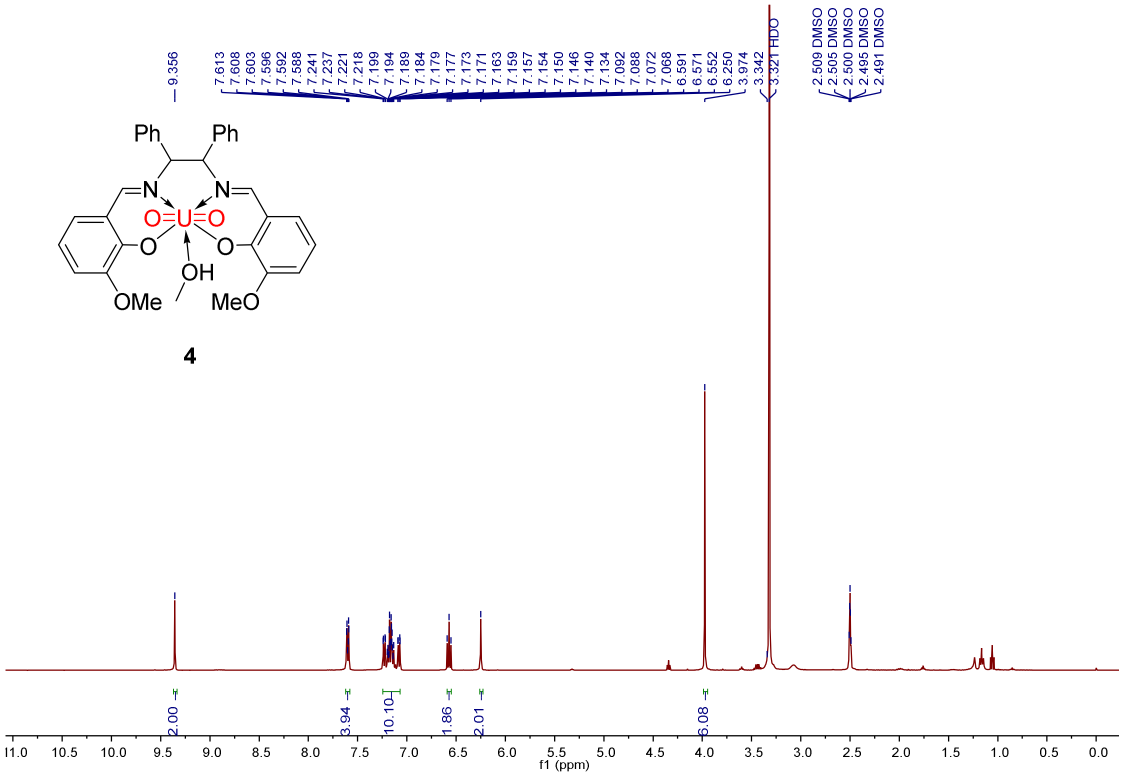

Figure S7. ^{1}H NMR spectrum of compound **4** (DMSO-d_6, 400 MHz)

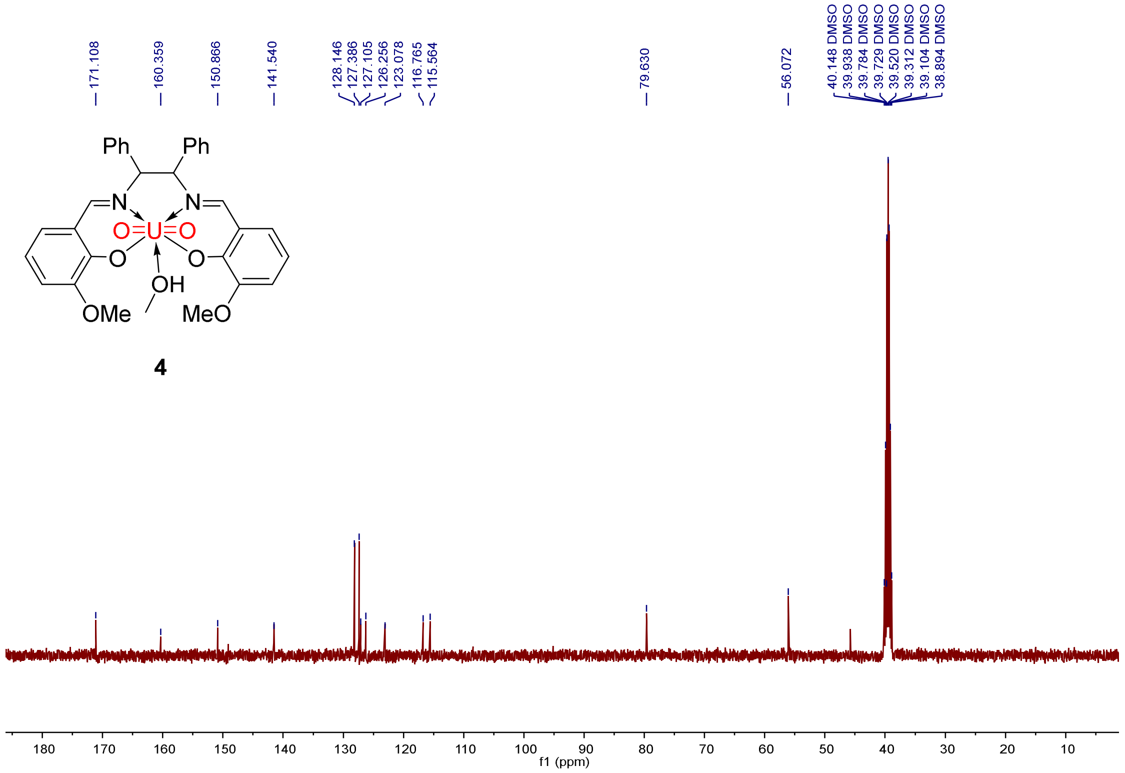

Figure S8. $^{13}C\{^{1}H\}$ NMR spectrum of compound **4** (DMSO-d_6, 100 MHz)

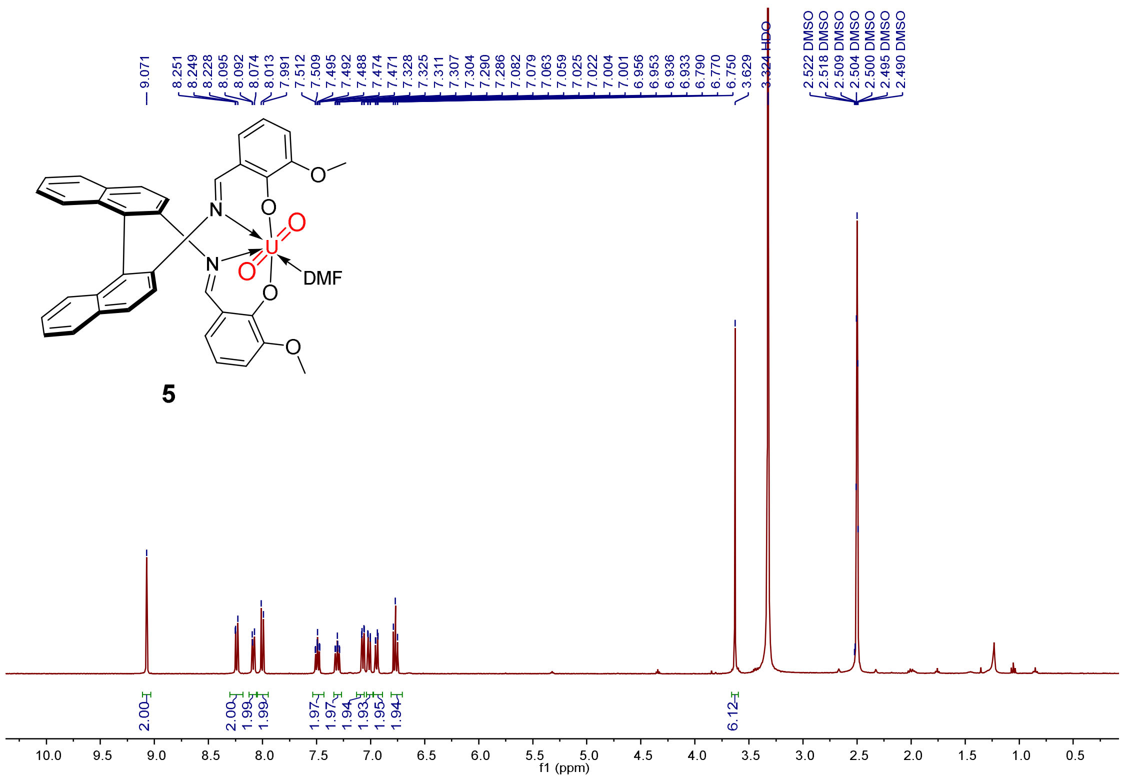

Figure S9. ^{1}H NMR spectrum of compound **5** (DMSO-d$_6$, 400 MHz)

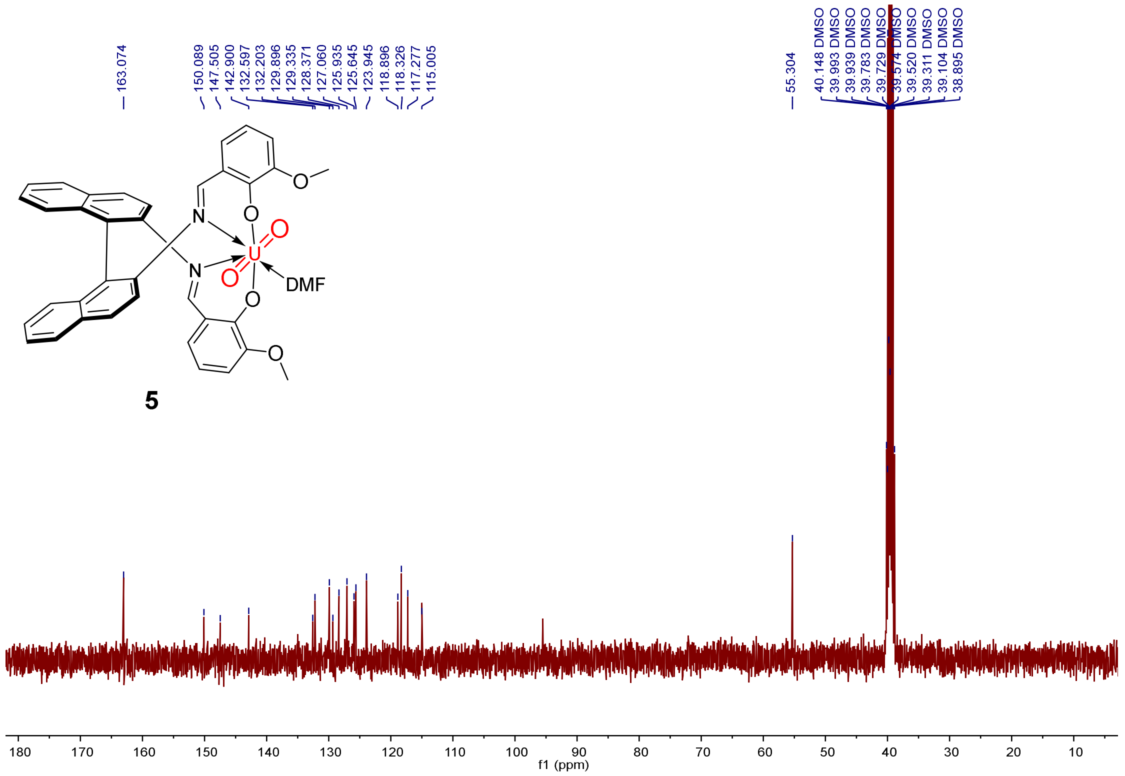

Figure S10. $^{13}C\{^{1}H\}$ NMR spectrum of compound **5** (DMSO-d$_6$, 100 MHz)

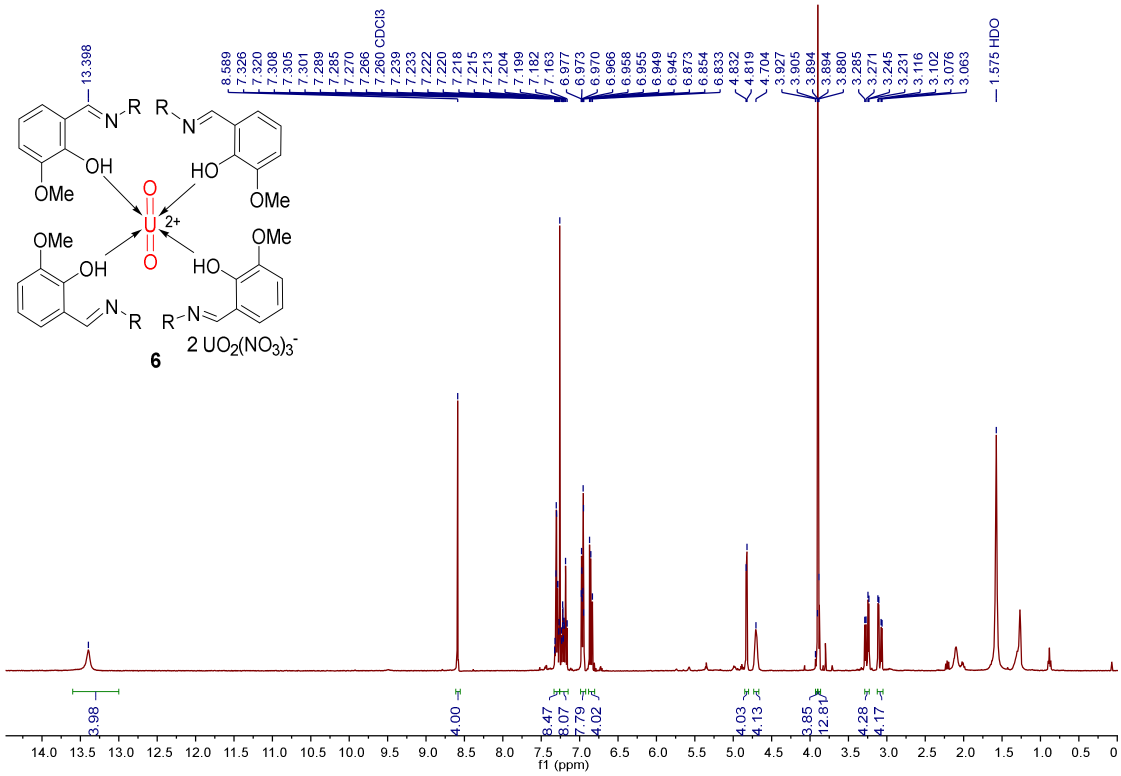

Figure S11. ^{1}H NMR spectrum of compound **6** (DMSO-d_6, 400 MHz)

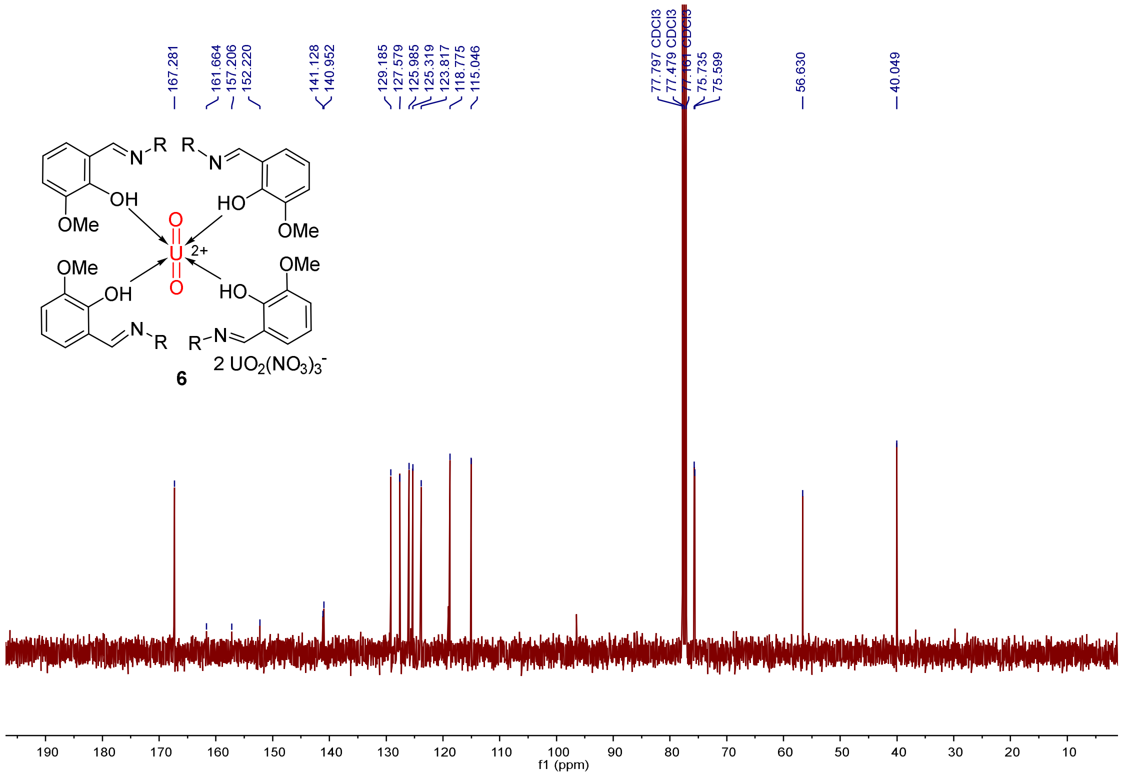

Figure S12. $^{13}C\{^{1}H\}$ NMR spectrum of compound **6** ($CDCl_3$, 100 MHz)

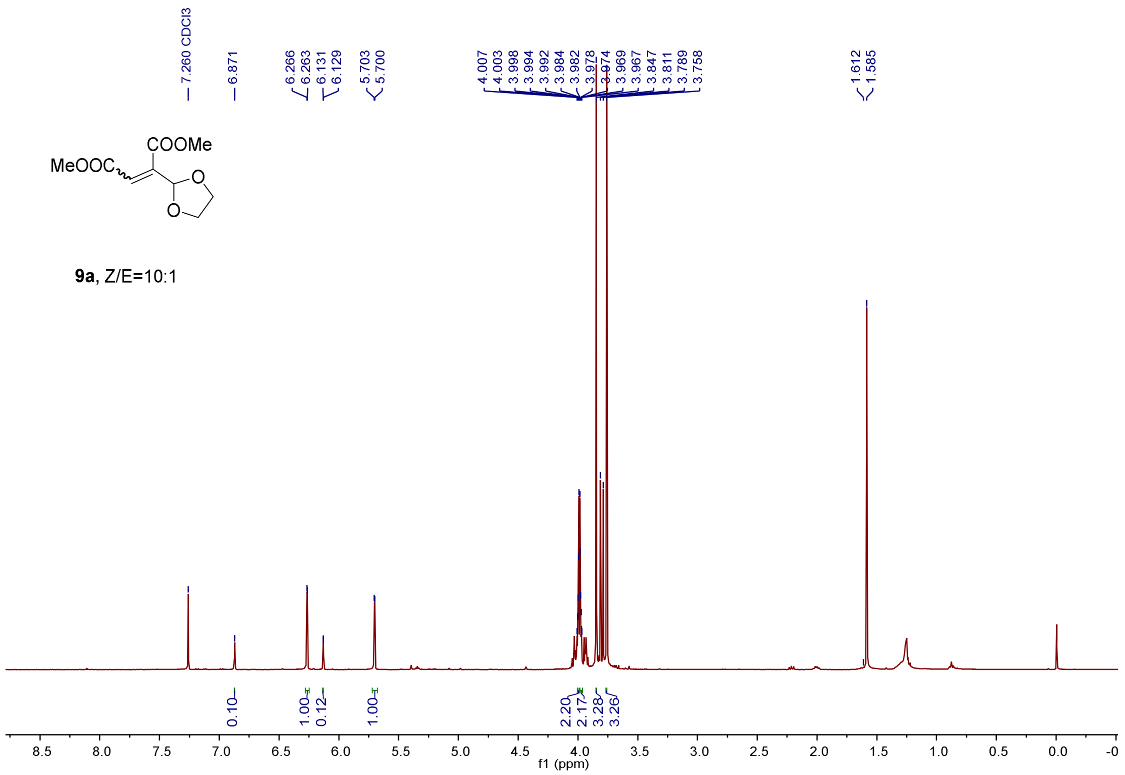

Figure S13. ^{1}H NMR spectrum of compound **9a** ($CDCl_3$, 400 MHz)

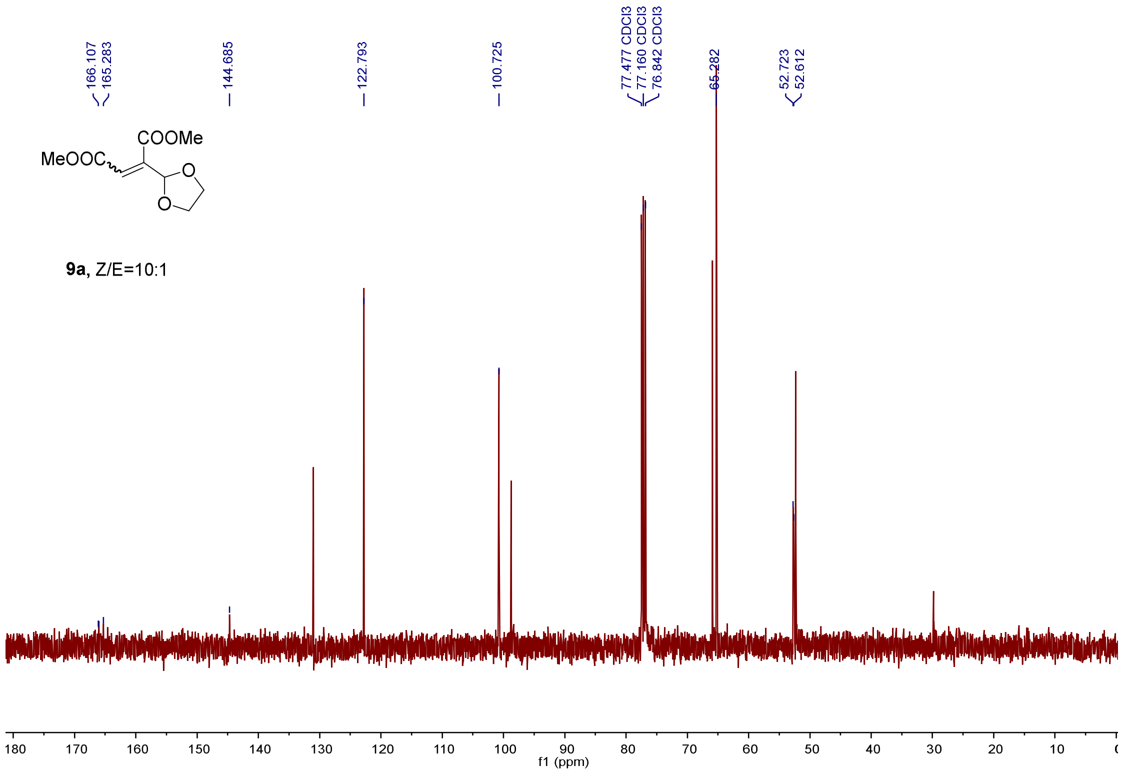

Figure S14. $^{13}C\{^{1}H\}$ NMR spectrum of compound **9a** ($CDCl_3$, 100 MHz)

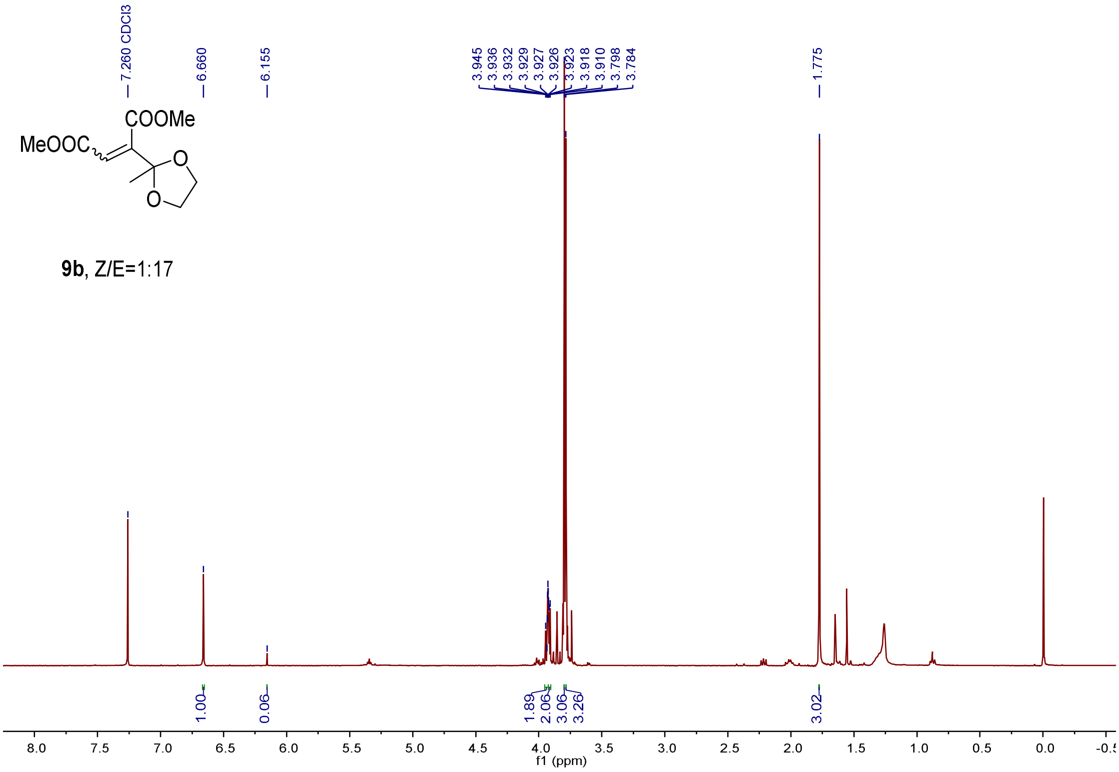

Figure S15. ^{1}H NMR spectrum of compound **9b** ($CDCl_3$, 400 MHz)

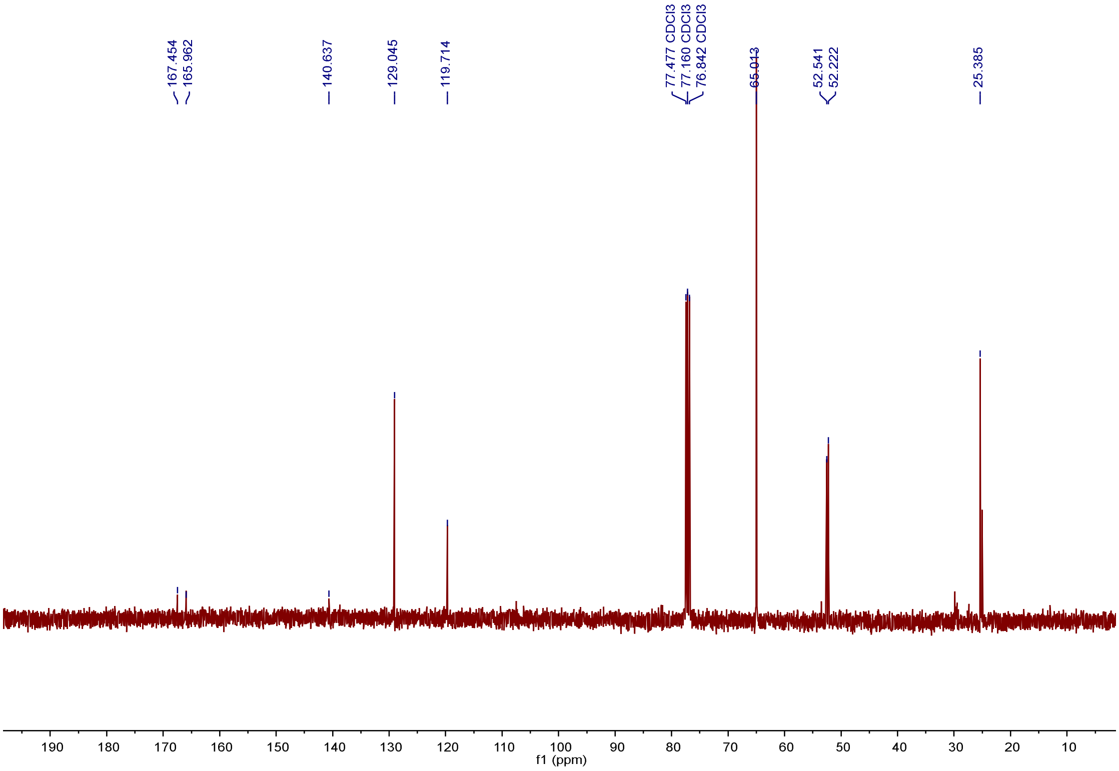

Figure S16. $^{13}C\{^{1}H\}$ NMR spectrum of compound **9b** ($CDCl_3$, 100 MHz)

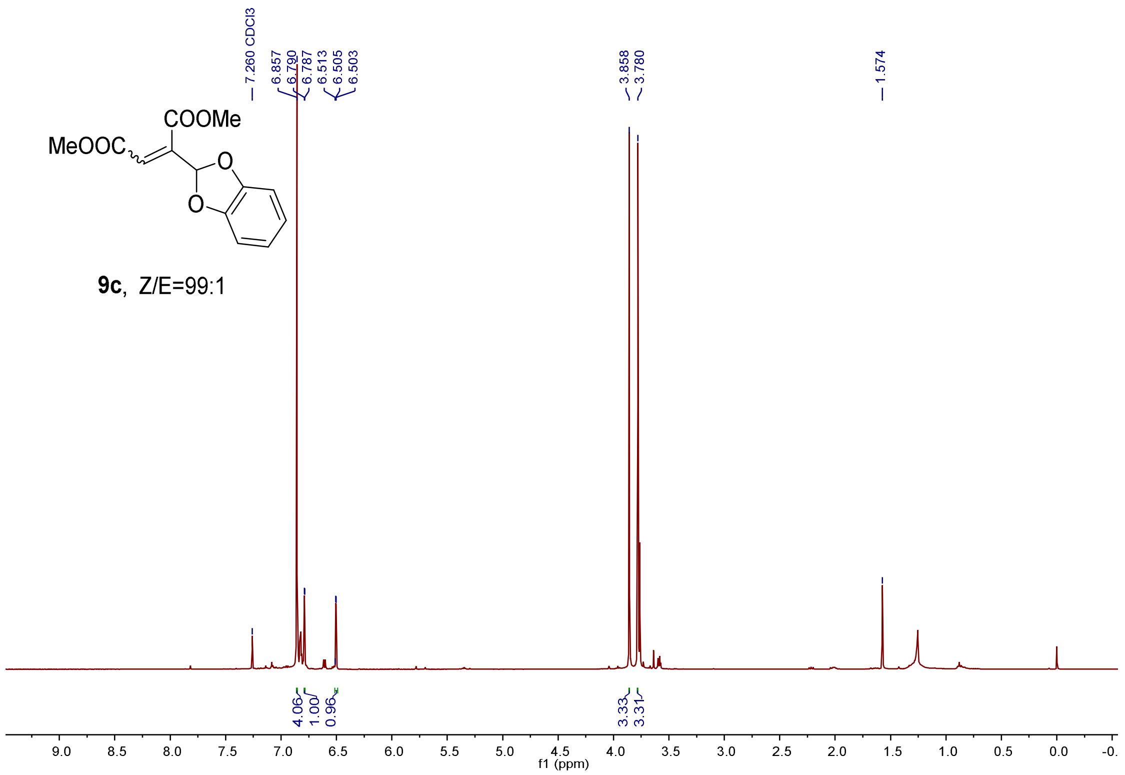

Figure S17. ^{1}H NMR spectrum of compound **9c** ($CDCl_3$, 400 MHz)

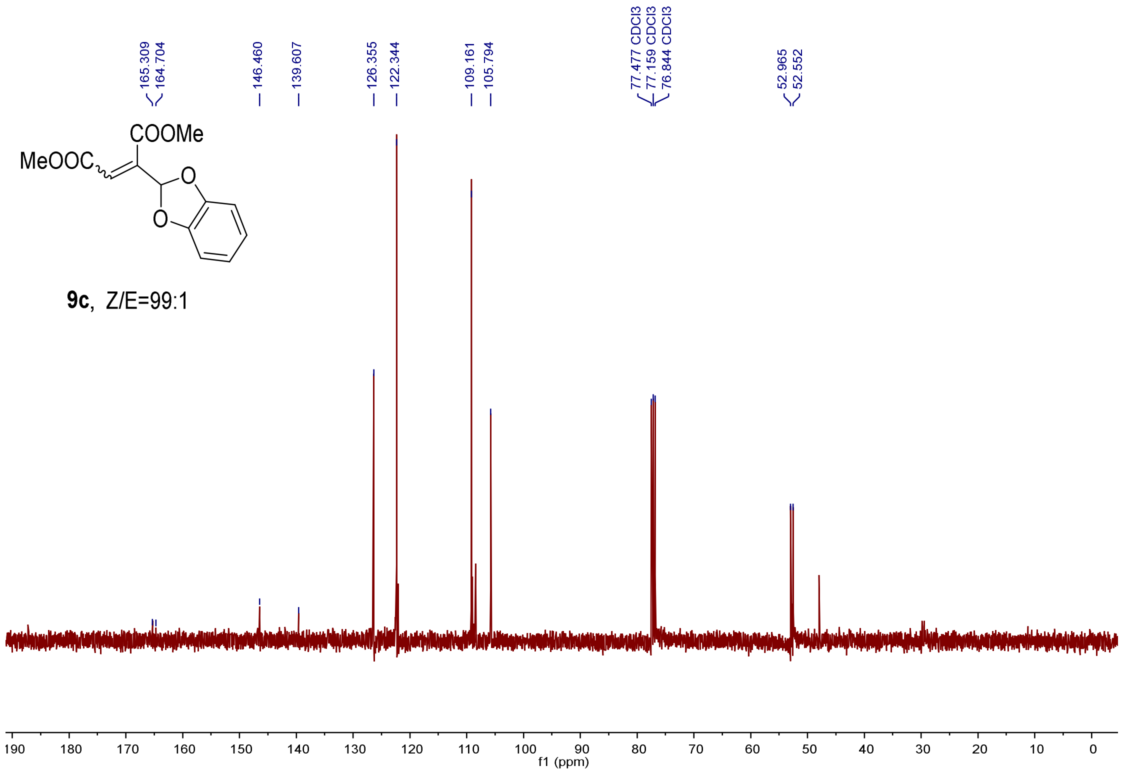

Figure S18. $^{13}C\{^{1}H\}$ NMR spectrum of compound **9c** ($CDCl_3$, 100 MHz)

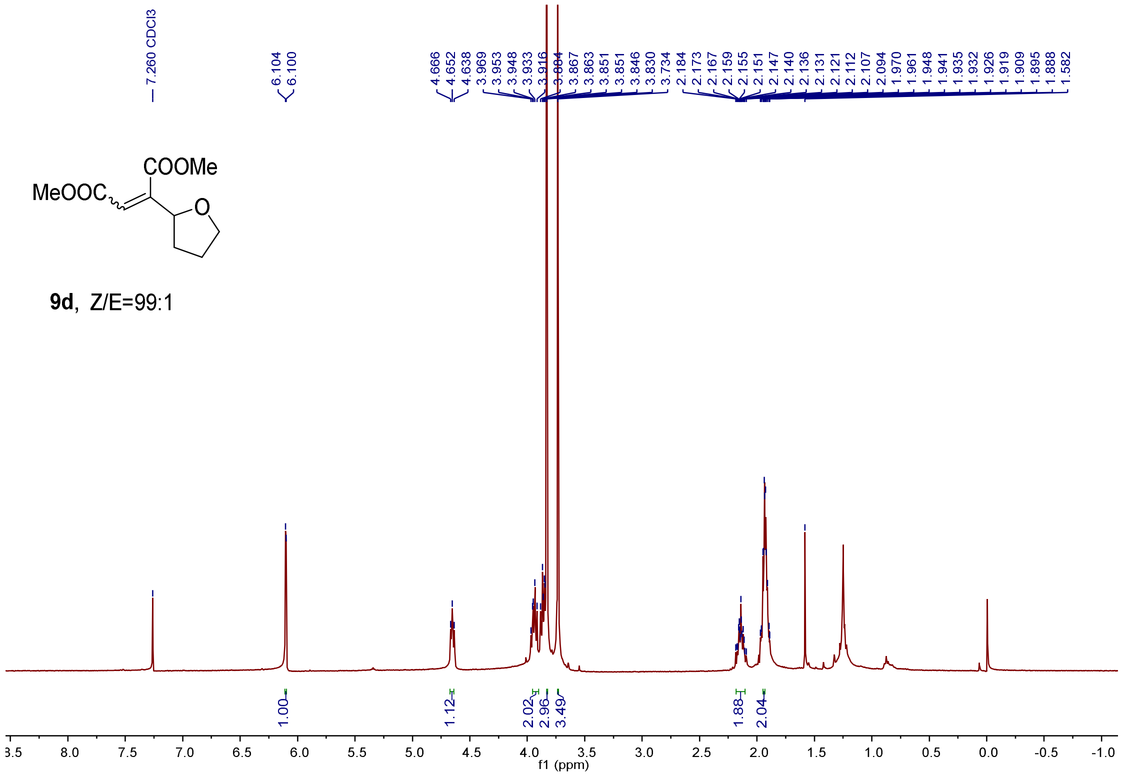

Figure S19. ^{1}H NMR spectrum of compound **9d** ($CDCl_3$, 400 MHz)

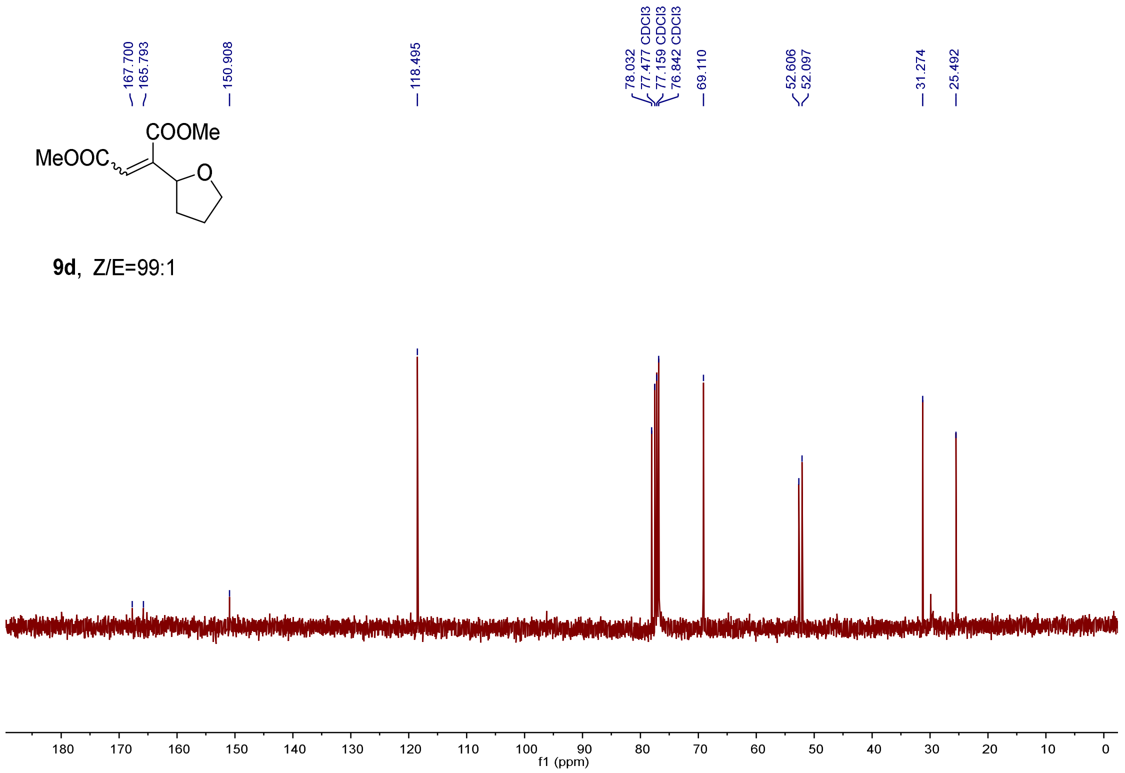

Figure S20. $^{13}C\{^{1}H\}$ NMR spectrum of compound **9d** ($CDCl_3$, 100 MHz)

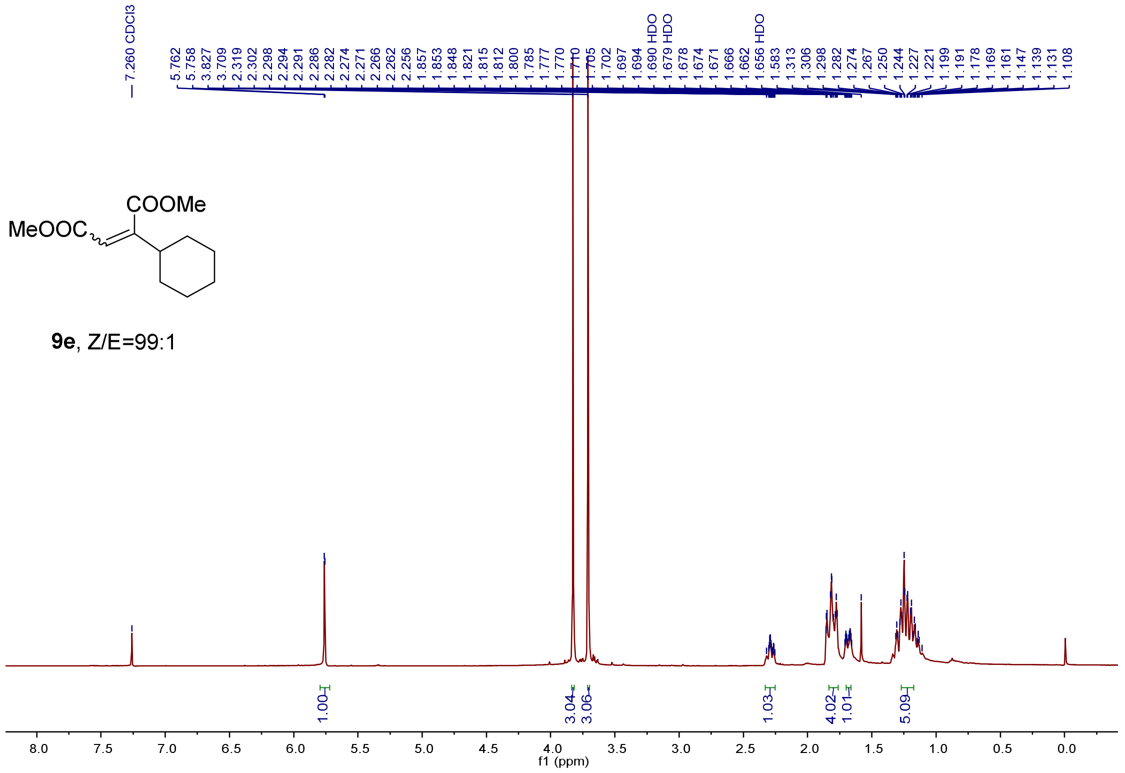

Figure S21. ^{1}H NMR spectrum of compound **9e** ($CDCl_3$, 400 MHz)

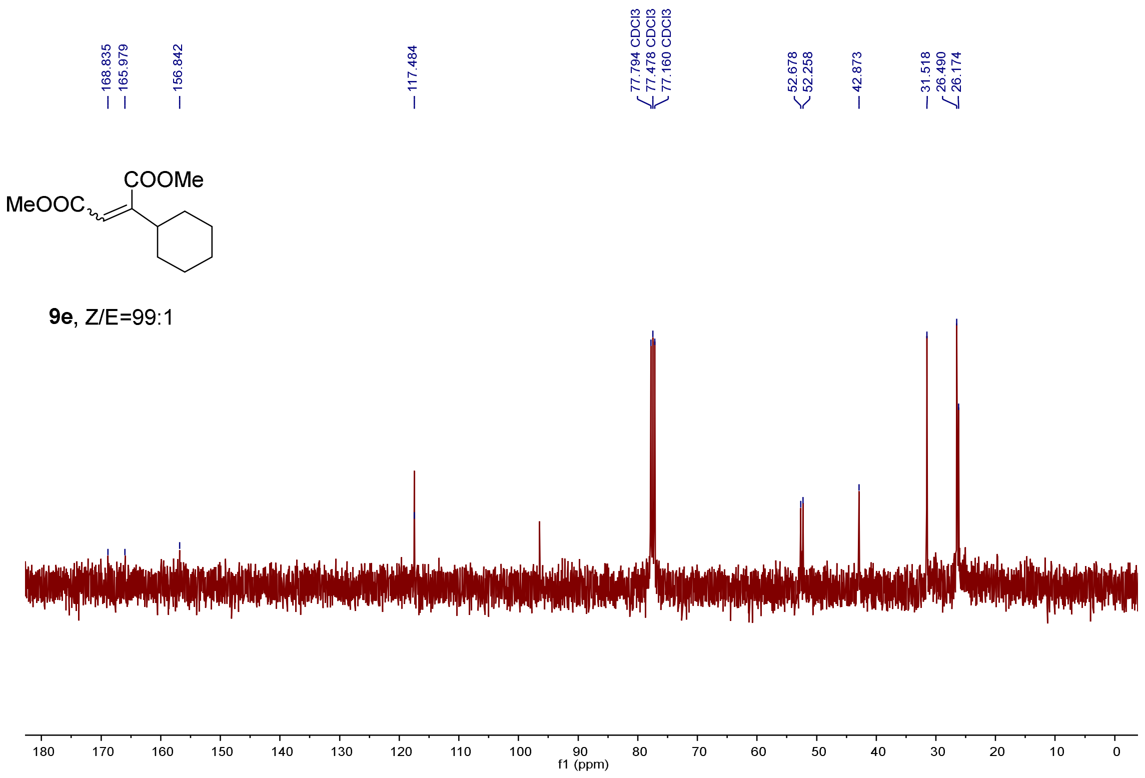

Figure S22. $^{13}C\{^{1}H\}$ NMR spectrum of compound **9e** ($CDCl_3$, 100 MHz)

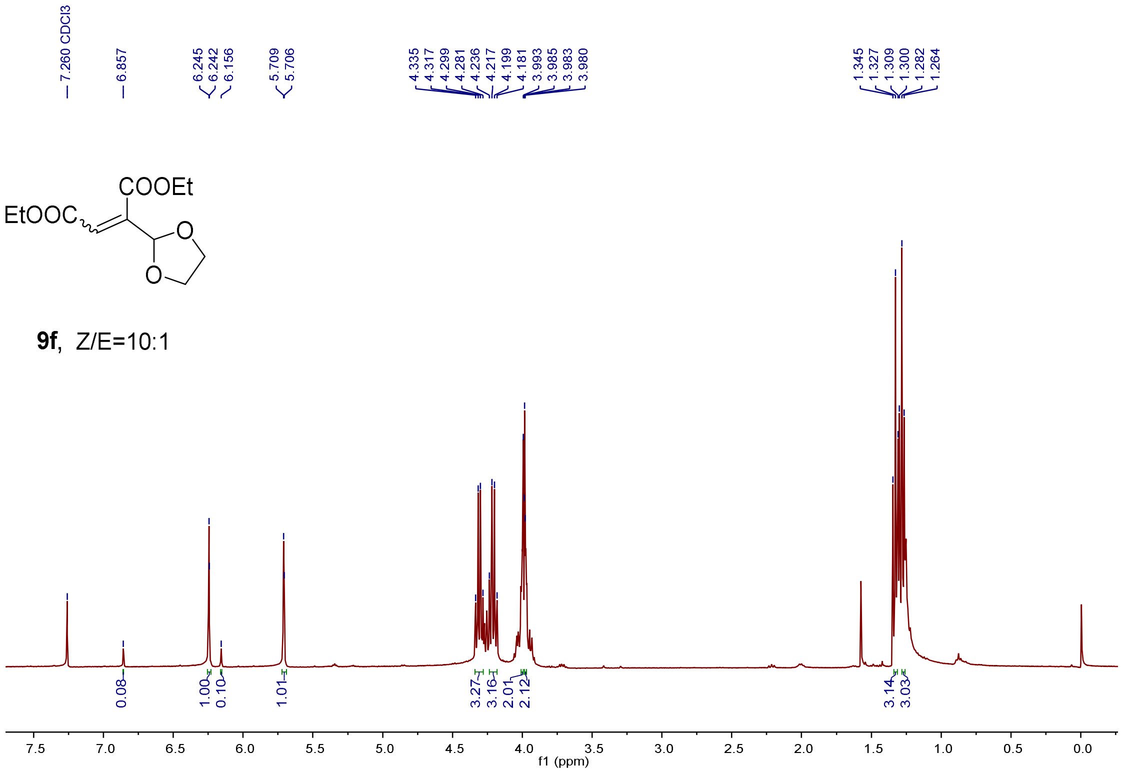

Figure S23. ^{1}H NMR spectrum of compound **9f** ($CDCl_3$, 400 MHz)

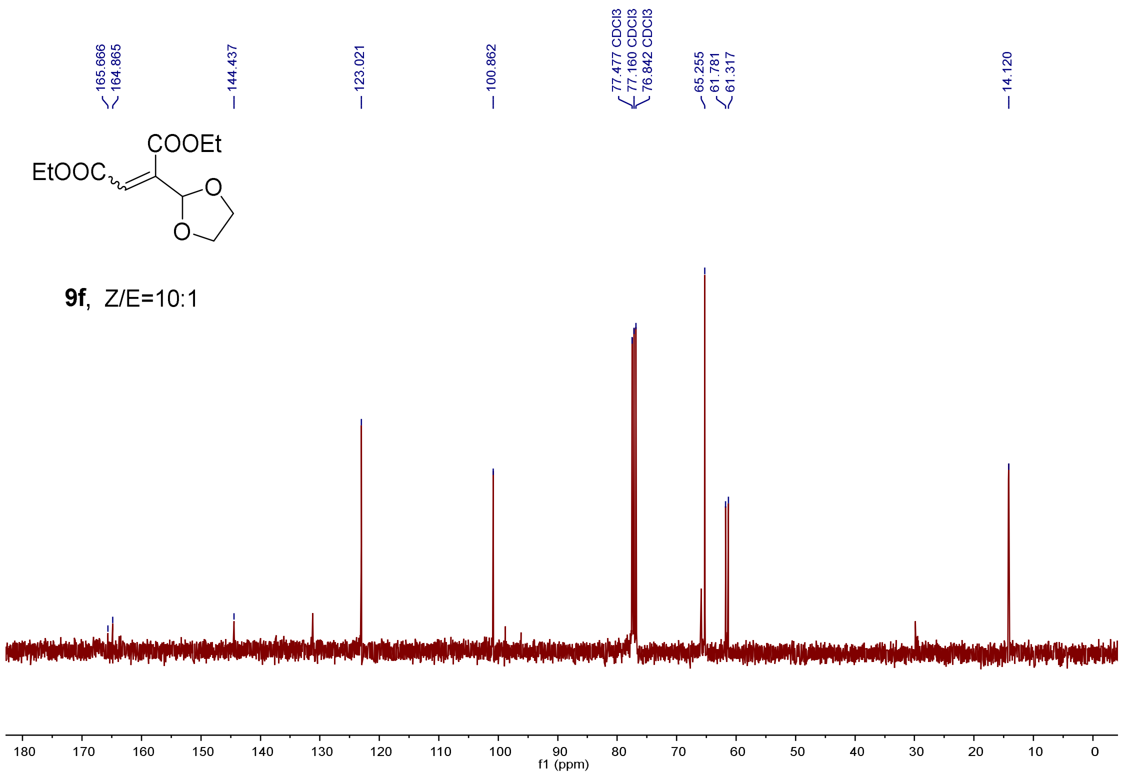

Figure S24. $^{13}C\{^{1}H\}$ NMR spectrum of compound **9f** ($CDCl_3$, 100 MHz)

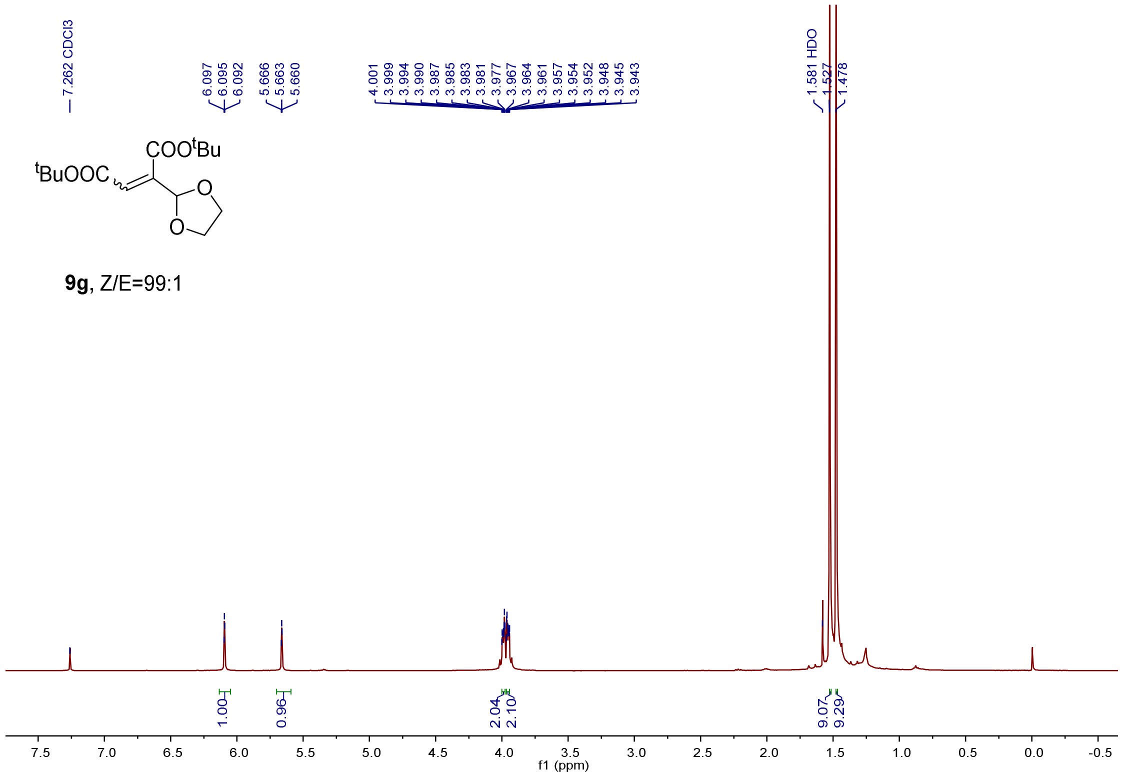

Figure S25. ^{1}H NMR spectrum of compound **9g** ($CDCl_3$, 400 MHz)

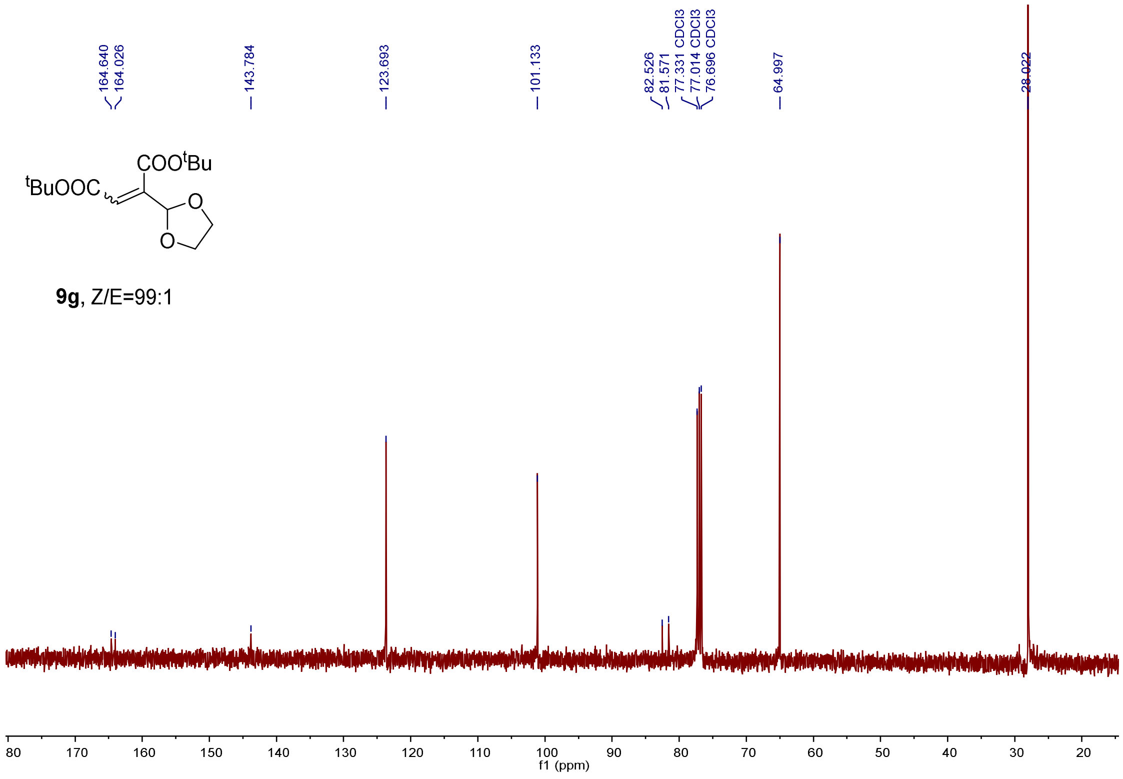

Figure S26. $^{13}C\{^1H\}$ NMR spectrum of compound **9g** ($CDCl_3$, 100 MHz)

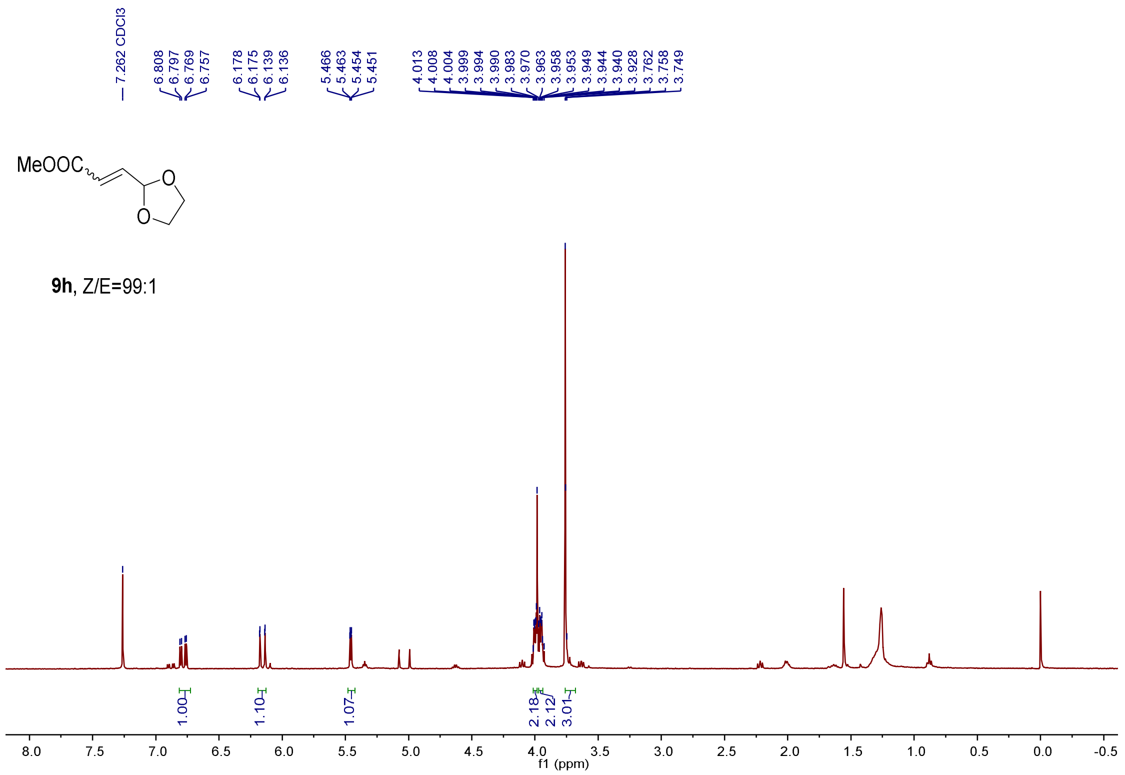

Figure S27. ^{1}H NMR spectrum of compound **9h** ($CDCl_3$, 400 MHz)

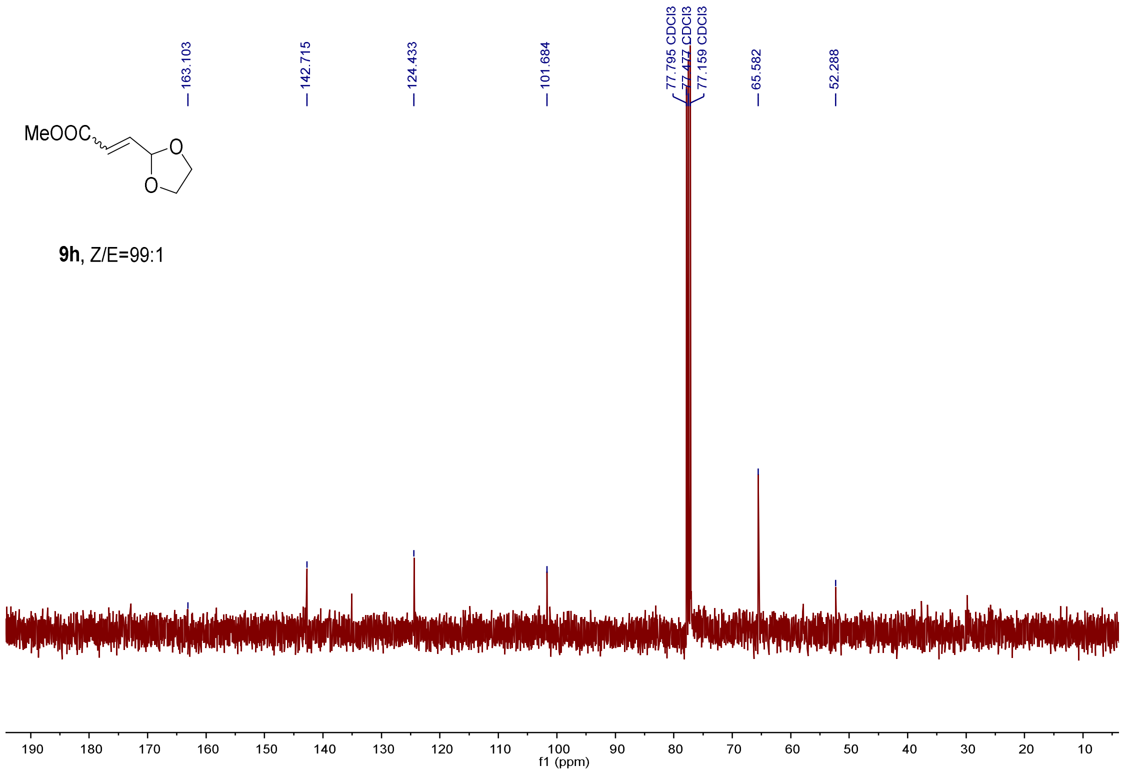

Figure S28. $^{13}C\{^{1}H\}$ NMR spectrum of compound **9h** ($CDCl_3$, 100 MHz)

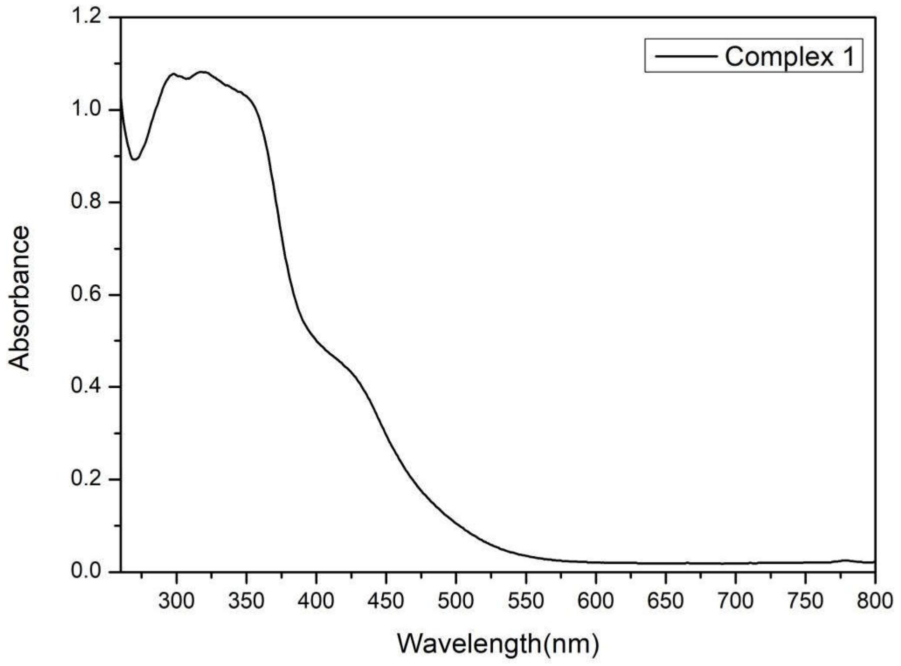

Figure S29. UV-visible absorption spectra for **1** measured in Dimethyl sulfoxide at room temperature

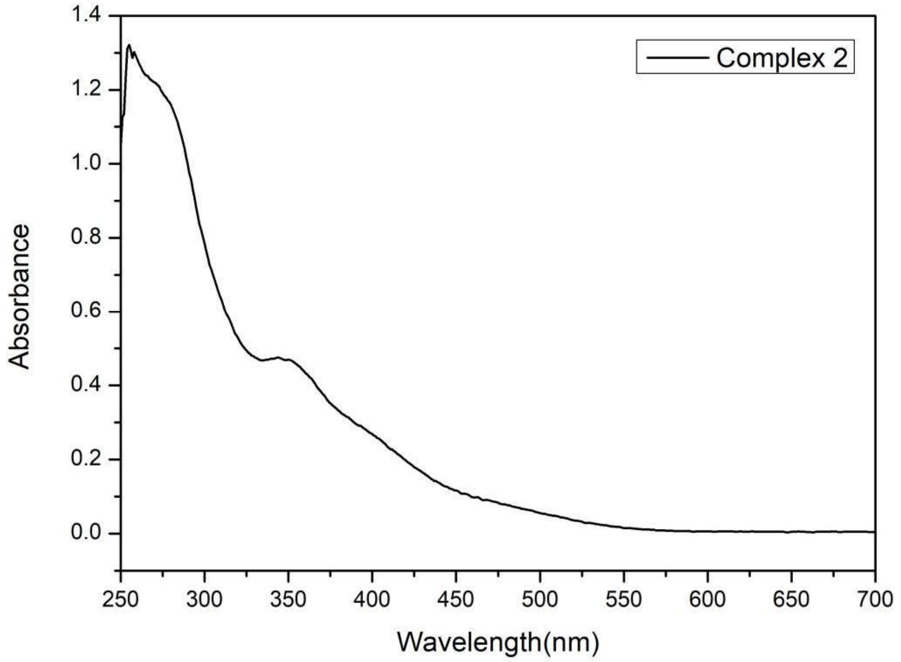

Figure S30. UV-visible absorption spectra for **2** measured in Dimethyl sulfoxide at room temperature

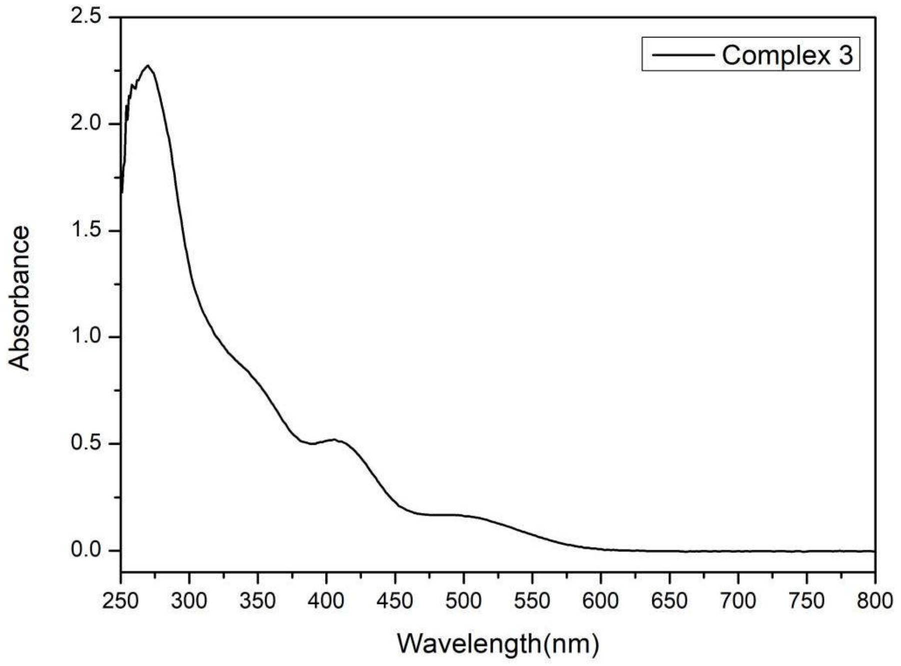

Figure S31. UV-visible absorption spectra for **3** measured in Dimethyl sulfoxide at room temperature

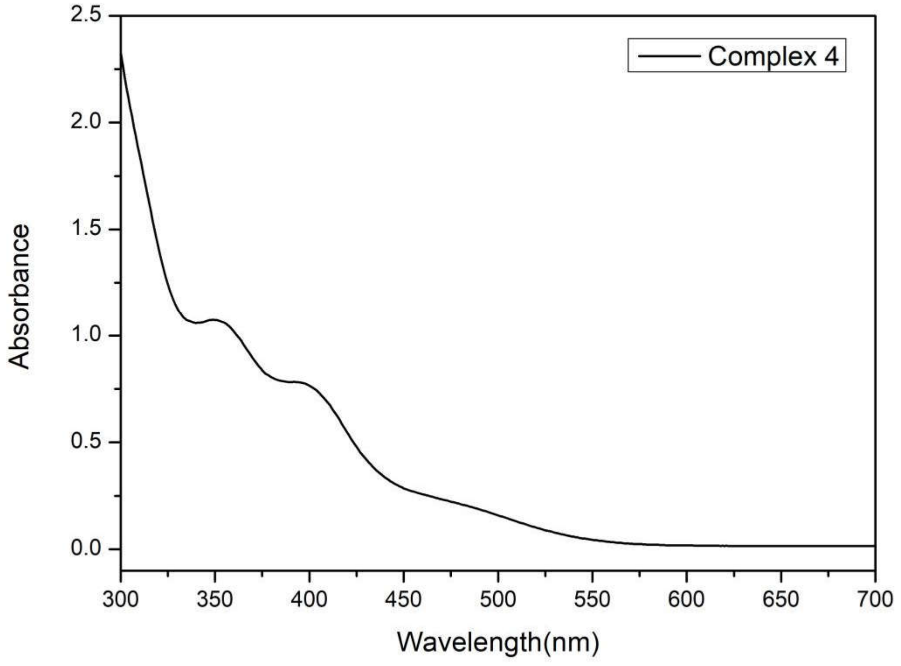

Figure S32. UV-visible absorption spectra for **4** measured in Dimethyl sulfoxide at room temperature

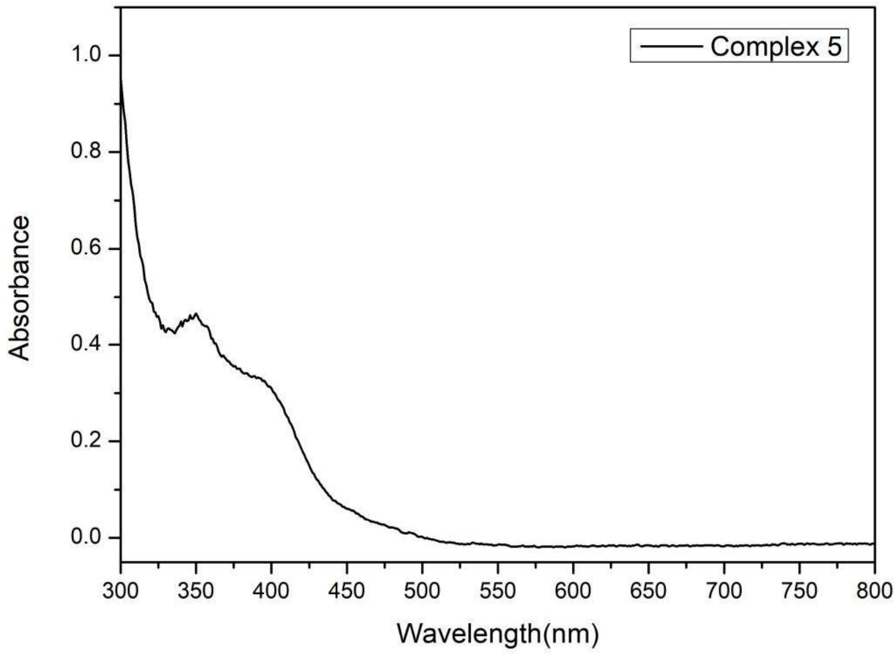

Figure S33. UV-visible absorption spectra for **5** measured in Dimethyl sulfoxide at room temperature

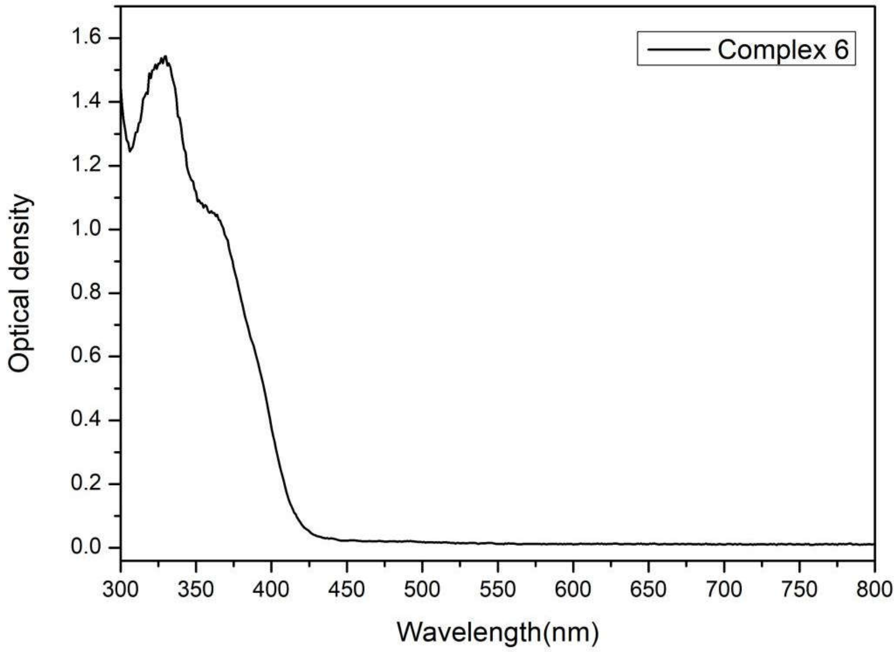

Figure S34. UV-visible absorption spectra for **6** measured in Dimethyl sulfoxide at room temperature

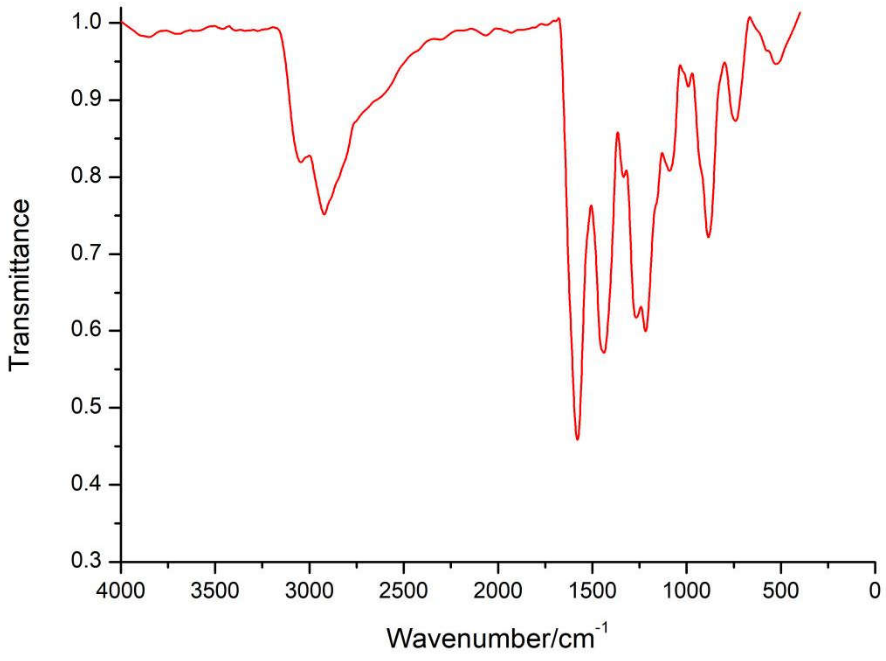

Figure S35. FT-IR spectrum of complex **1**

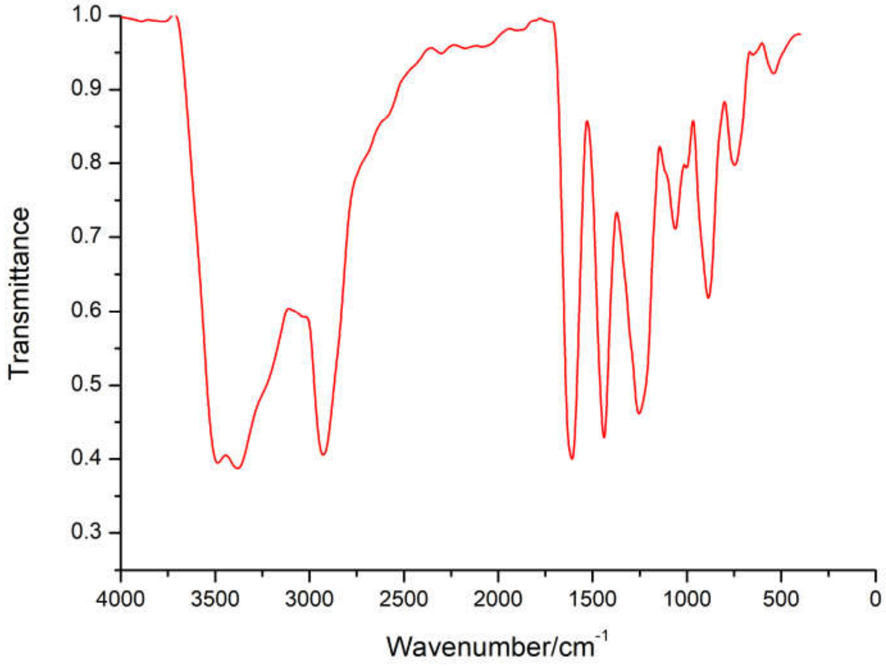

Figure S36. FT-IR spectrum of complex **2**

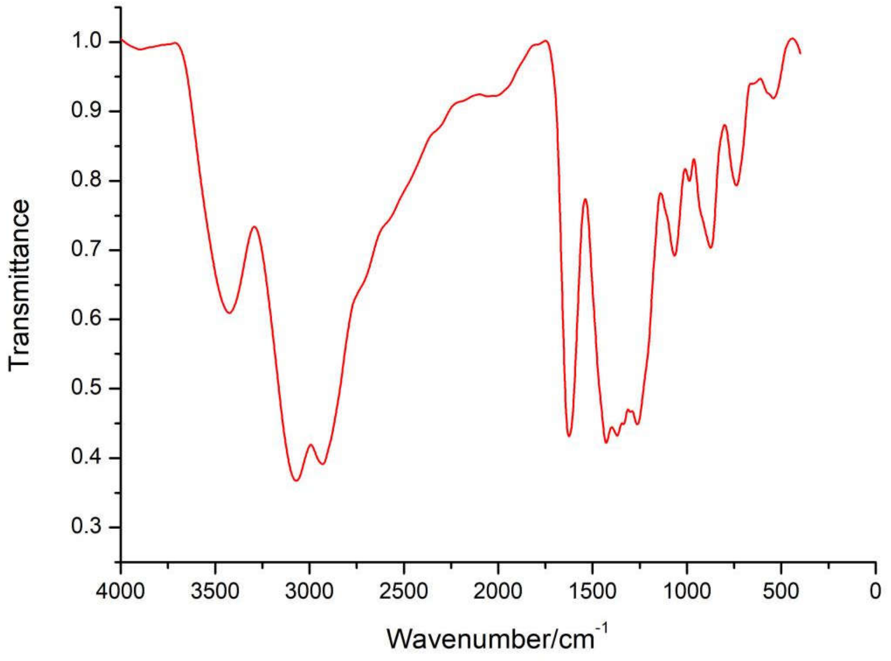

Figure S37. FT-IR spectrum of complex **3**

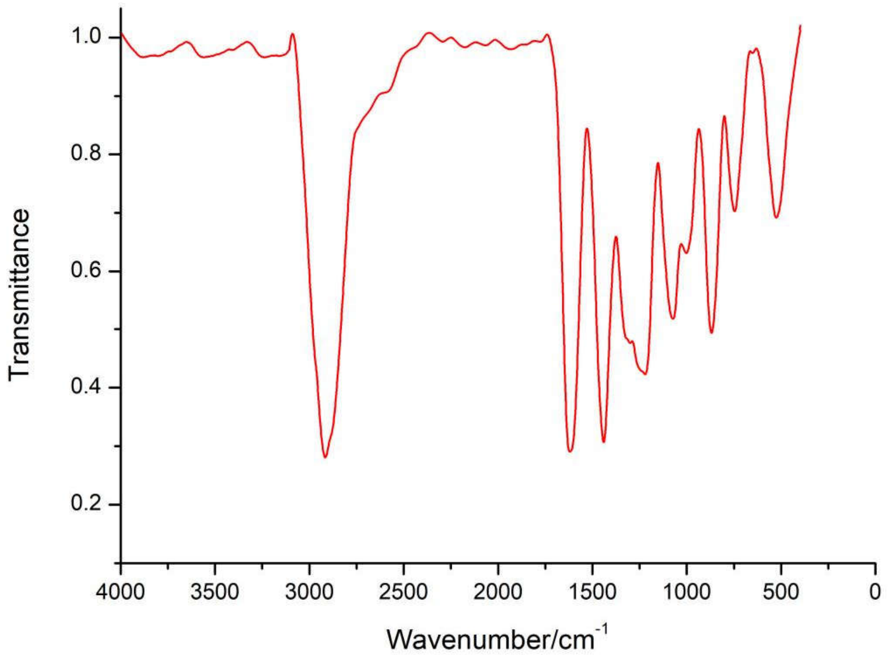

Figure S38. FT-IR spectrum of complex **4**

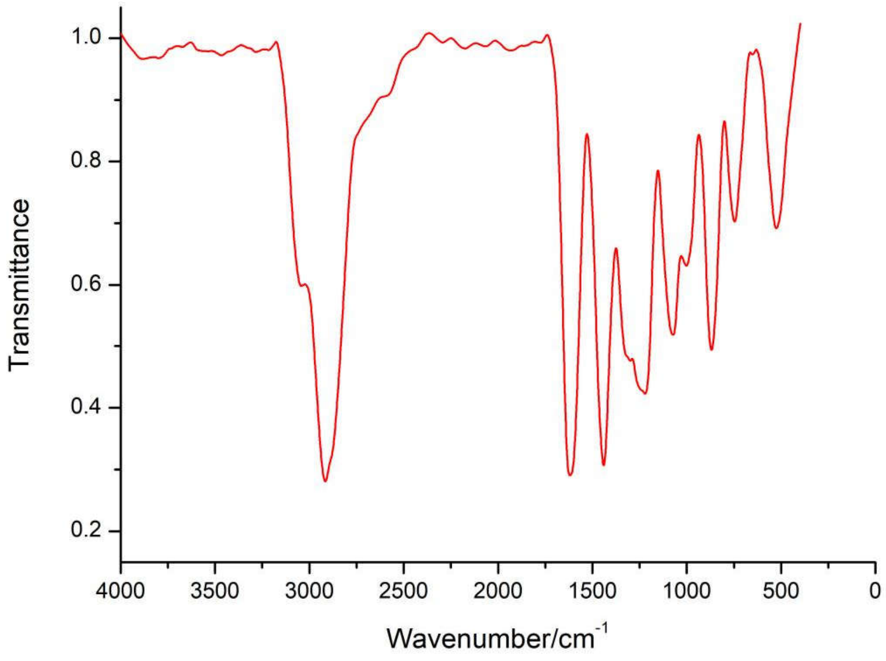

Figure S39. FT-IR spectrum of complex **5**

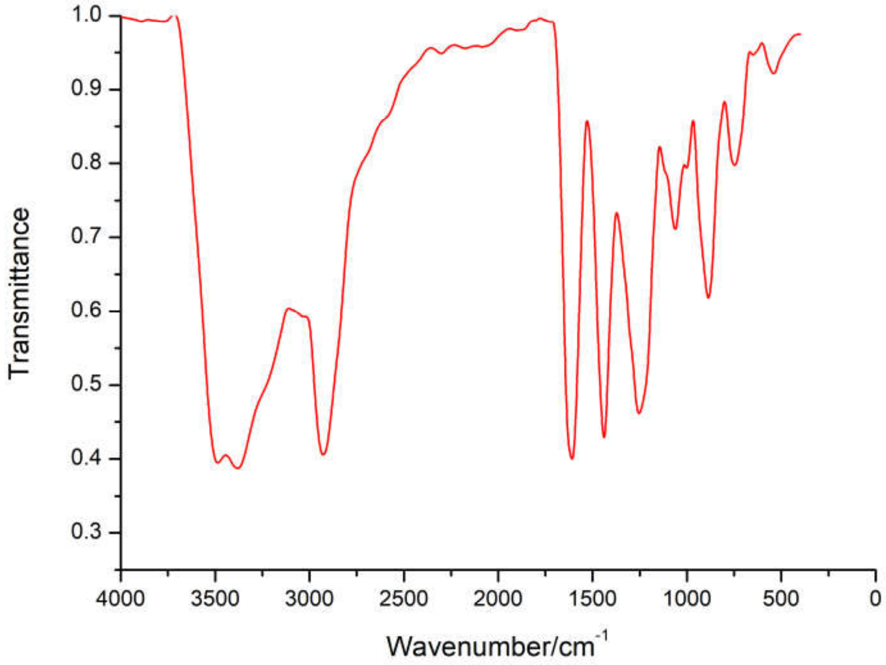

Figure S40. FT-IR spectrum of complex **6**

2. X-ray crystallographic analysis

Single-crystal X-ray diffraction data for complexes **1**, **2**, **3**, **4**, **5**, and **6** were collected on BRUKER D8 VENTURE PHOTON II detector with a radiation source of Mo(Kα) (0.71073 Å) or Ga(Kα) (1.34139 Å). All structures were solved by Patterson methods and refined on F2 using full-matrix least-squares methods with SHELXTL-2014 program package. All non-hydrogen atoms were refined on F2 by full-matrix least-squares procedures with the use of anisotropic displacement parameters. Hydrogen atoms were introduced at their geometric positions and refined as riding atoms. CCDC-2350338 (**1**), 2350339 (**2**), 2350340 (**3**), 2350342 (**4**), 2350341 (**5**), and 2350343 (**6**) contain the crystallographic data for this paper. These data can be obtained free of charge from The Cambridge Crystallographic Data Centre via www.ccdc.cam.ac.uk/data-request/cif. Details regarding the data collection and refinement for these complexes were given in Tables S1-S2.

Table S1. Crystal data and structural refinement for **1**, **2**, and **3**.

Complex	**1**	**2**	**3**
formula	$C_{28}H_{32}N_4O_8U$	$C_{24}H_{32}N_2O_8U$	$C_{31}H_{50}N_8O_{12}U$
Mr [g/mol]	790.617	714.54	964.82
Temp./K	296.15	170	153.0
Crystal system	orthorhombic	Monoclinic	Monoclinic
Space group	Pbca	$P2_1/n$	$P2_1/n$
a [Å]	20.5813(14)	11.909(3)	17.848(4)
b [Å]	8.7262(5)	9.053(2)	11.210(2)
c [Å]	32.178(2)	23.779(5)	20.095(4)
α [°]	90	90	90.00(3)
β [°]	90	91.205(10)	105.75(3)
γ [°]	90	90	90.00(3)
Volume [Å^3]	5779.1(6)	2563.1(10)	3869.6(15)
Z	8	4	4
ρ_{calc}g [cm^3]	1817	1.849	1.656
μ [mm^{-1}]	5.672	6.382	8.926
F(000)	2997.3	1380.0	1920.0
Crystal size [mm^3]	0.23 × 0.21 × 0.2	0.21 × 0.2 × 0.2	0.21 × 0.2 × 0.2
Radiation	MoKα (λ = 0.71073)	MoKα (λ = 0.71073)	GaKα (λ = 1.34139)

2Θ range for data collection [°]	4.7 to 55	3.794 to 55.006	9.628 to 108.132
Index ranges	-26 ≤ h ≤ 23,<br>-11 ≤ k ≤11,<br>-41 ≤ l ≤38	-15 ≤ h ≤ 15,<br>-11≤ k ≤ 11,<br>-30 ≤ l ≤ 30	-21 ≤ h ≤21,<br>-12 ≤k ≤ 13,<br>-24 ≤ l ≤ 24
Reflections collected	51937	75763	42587
Independent reflections	6624<br>[R_{int} = 0.0786]	5864<br>[R_{int} = 0.1089]	7030<br>[R_{int} = 0.0542]
Data/restraints/parameters	6624/0/376	5864/42/324	7030/0/478
Goodness-of-fit on F^2	1.058	1.274	1.070
Final R indexes [I>=2$\sigma(I)$]	R_1 = 0.0267,<br>wR_2 = 0.0599	R_1 = 0.0742,<br>wR_2 = 0.1850	R_1 = 0.0259,<br>wR_2 = 0.0684
Final R indexes [all data]	R_1 = 0.0418,<br>wR_2 = 0.0678	R_1 = 0.0802,<br>wR_2 = 0.1934	R_1 = 0.0268,<br>wR_2 = 0.0690
Largest diff. peak/hole / e Å^{-3}	0.92/-1.12	1.68/-4.92	0.79/-1.28

Table S2. Crystal data and structural refinement for **4**, **5**, and **6**.

Complex	**4**	**5**	**6**
formula	$C_{34}H_{40}N_2O_9U$	$C_{39}H_{33}N_4O_6U$	$C_{71}H_{71}N_{10}O_{36}U_3$
Mr [g/mol]	858.71	858.71	2673.51
Temp./K	193.15	296.15	193.0
Crystal system	orthorhombic	orthorhombic	tetragonal
Space group	*Pbca*	$P2_12_12_1$	$P4_3$
a [Å]	13.9886(5)	11.4183(3)	18.323(2)
b [Å]	18.1412(6)	17.0309(5)	18.323
c [Å]	26.0453(9)	17.7483(5)	27.436(4)
α [°]	90	90	90
β [°]	90	90	90
γ [°]	90	90	90
Volume [Å^3]	6609.5(4)	3451.40(17)	9211(3)
Z	8	4	4
ρ_{calc}g [cm^3]	1.726	1.653	1.928
μ [mm^{-1}]	10.691	4.754	12.752
F(000)	3376.0	1609.0	5136.0
Crystal size [mm^3]	0.20 × 0.18 × 0.16	0.21× 0.20× 0.20	0.2× 0.2× 0.18
Radiation	GaKα (λ = 1.34139)	MoKα (λ = 0.71073)	GaKα (λ = 1.34139)
2Θ range for data collection [°]	5.904 to 105.958	4.242 to 55.06	4.196-104.184
Index ranges	-16≤h ≤16,<br>-18≤ k ≤21,<br>-30≤ l≤ 31	-14≤ h ≤ 12,<br>-22 ≤ k ≤ 22,<br>-22 ≤ l ≤ 23	-21≤ h ≤ 21,<br>-21≤ k≤ 21,<br>-32 ≤ l ≤ 32
Reflections collected	70240	31298	90532
Independent reflections	5836	7920	15666

	[R_{int} = 0.0837]	[R_{int} = 0.0497]	[R_{int} = 0.0839]
Data/restraints/parameters	5836/828/517	7920/0/205	15666/1646/1163
Goodness-of-fit on F^2	1.416	1.277	1.028
Final R indexes [I>=2σ(I)]	R_1 = 0.0803, wR_2 = 0.1829	R_1 = 0.0342, wR_2 = 0.0777	R_1 = 0.0539, wR_2 = 0.1296
Final R indexes [all data]	R_1 = 0.0862, wR_2 = 0.1848	R_1 = 0.0389, wR_2 = 0.0792	R_1 = 0.0871, wR_2 = 0.1470
Largest diff. peak/hole / e Å^{-3}	2.54/-3.83	1.20/-0.98	2.21/-1.15

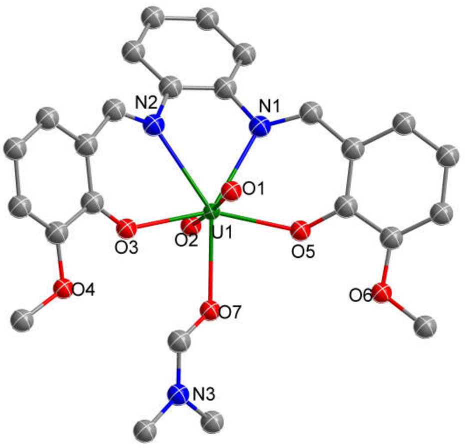

Figure S41. Solid-state structure of **1** by X-ray crystallography with 50% probability ellipsoids. All the hydrogen atoms were omitted for clarity.

Table S3. Selected bond distances and angles for complex **1**.

Bond Distances(Å)					
N1-U1	2.538(3)	O2-U1	1.782(3)	O7-U1	2.434(3)
N2-U1	2.535(3)	O3-U1	2.246(3)		
O1-U1	1.793(3)	O5-U1	2.272(2)		
Bond Angles(°)					
N1-U1-N2	63.89(9)	N2-U1-O3	70.35(10)	O5-U1-O7	75.66(9)
N1-U1-O5	70.88(9)	O3-U1-O7	80.16(10)		

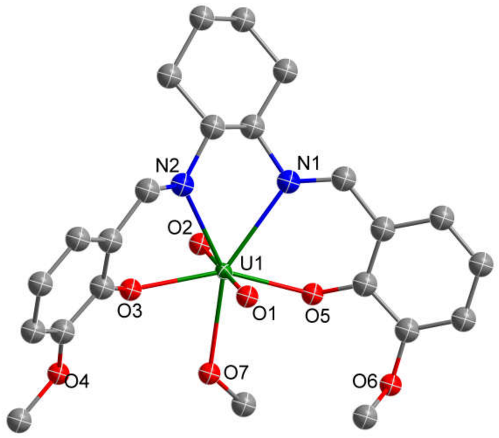

Figure S42. Solid-state structure of **2** by X-ray crystallography with 50% probability ellipsoids. All the hydrogen atoms were omitted for clarity.

Table S4. Selected bond distances and angles for complex **2**.

Bond Distances(Å)					
N1-U1	2.542(10)	O2-U1	1.791(8)	O7-U1	2.513(8)
N2-U1	2.575(9)	O3-U1	2.250(8)		
O1-U1	1.795(8)	O5-U1	2.248(8)		
Bond Angles(°)					
N1-U1-N2	64.7(3)	N2-U1-O3	70.6(3)	O5-U1-O7	70.7(3)
N1-U1-O5	70.7(3)	O3-U1-O7	77.4(3)		

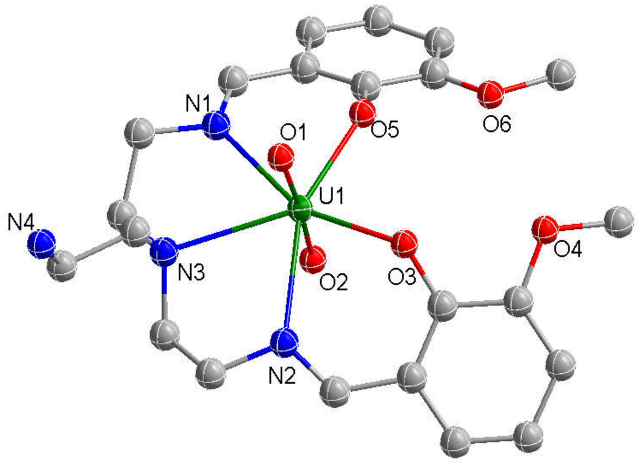

Figure S43. Solid-state structure of **3** by X-ray crystallography with 50% probability ellipsoids. All the hydrogen atoms and Nitrate were omitted for clarity.

Table S5. Selected bond distances and angles for complex **3**.

Bond Distances(Å)					
N1-U1	2.571(3)	O1-U1	1.776(3)	O6-U1	2.261(3)
N2-U1	2.544(3)	O2-U1	1.765(3)		
N3-U1	2.665(2)	O3-U1	2.222(2)		
Bond Angles(°)					
N1-U1-N3	66.63(10)	N2-U1-N3	67.00(9)	O3-U1-O6	85.20(10)
N1-U1-O6	71.96(10)	N2-U1-O3	71.42(10)		

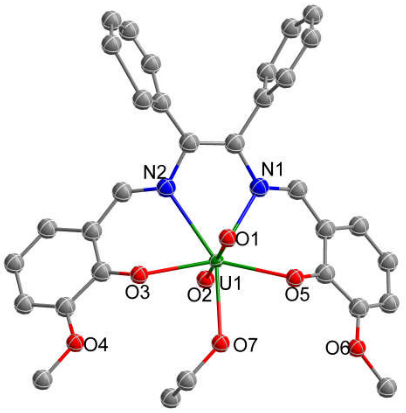

Figure S44. Solid-state structure of **4** by X-ray crystallography with 50% probability ellipsoids. All the hydrogen atoms were omitted for clarity.

Table S6. Selected bond distances and angles for complex **4**.

Bond Distances(Å)					
N1-U1	2.498(13)	O2-U1	1.776(11)	O7-U1	2.44(4)
N2-U1	2.552(12)	O3-U1	2.289(10)		
O1-U1	1.767(10)	O5-U1	2.272(9)		
Bond Angles(°)					
N1-U1-N2	63.2(4)	N2-U1-O3	71.4(4)	O5-U1-O7	77.1(10)
N1-U1-O5	69.3(4)	O3-U1-O7	80.6(11)		

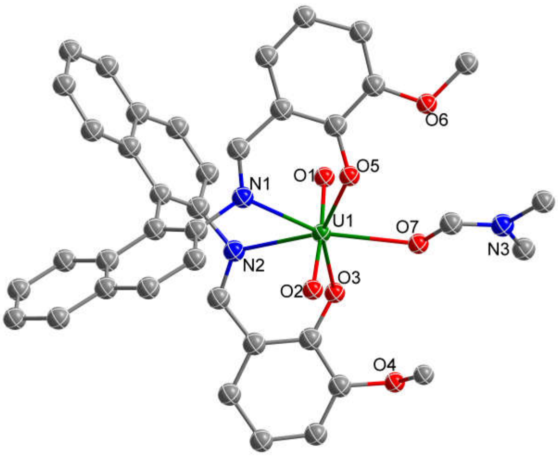

Figure S45. Solid-state structure of **5** by X-ray crystallography with 50% probability ellipsoids. All the hydrogen atoms were omitted for clarity.

Table S7. Selected bond distances and angles for complex **5**.

Bond Distances(Å)					
N1-U1	2.578(5)	O2-U1	1.782(4)	O7-U1	2.419(4)
N2-U1	2.562(4)	O3-U1	2.267(4)		
O1-U1	1.783(4)	O5-U1	2.272(4)		
Bond Angles(°)					
N1-U1-N2	70.09(14)	N2-U1-O3	69.13(14)	O5-U1-O7	74.23(14)
N1-U1-O5	68.48(15)	O3-U1-O7	79.42(16)		

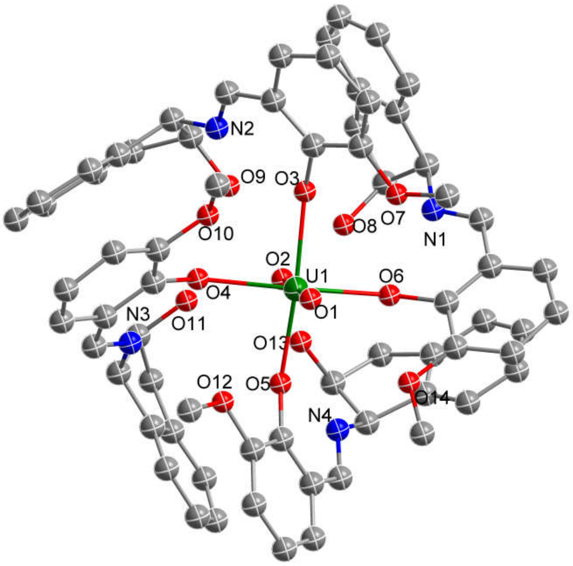

Figure S46. Solid-state structure of **6** by X-ray crystallography with 50% probability ellipsoids. All the hydrogen atoms and Nitrate were omitted for clarity.

Table S8. Selected bond distances and angles for complex **6**.

Bond Distances(Å)					
O1-U1	1.761(15)	O2-U1	1.746(15)	O3-U1	2.263(9)
O4-U1	2.284(10)	O5-U1	2.286(10)	O6-U1	2.285(10)
Bond Angles(º)					
O3-U1-O4	88.0(4)	O4-U1-O5	90.8(4)	O5-U1-O6	87.1(4)
O6-U1-O3	89.4(4)				
